# Antimicrobial Resistance in the Terrestrial Environment of Agricultural Landscapes in Norway

**DOI:** 10.3390/microorganisms12091854

**Published:** 2024-09-06

**Authors:** Live L. Nesse, Kristin Forfang, Jannice Schau Slettemeås, Snorre Hagen, Marianne Sunde, Abdelhameed Elameen, Gro Johannessen, Marianne Stenrød, Girum Tadesse Tessema, Marit Almvik, Hans Geir Eiken

**Affiliations:** 1 Department of Food Safety and Animal Health Research, Norwegian Veterinary Institute, N-1431 Ås, Norway; live.nesse@vetinst.no (L.L.N.); jannice.schau-slettemeas@vetinst.no (J.S.S.); gro.johannessen@vetinst.no (G.J.); 2 Division of Environment and Natural Resources, Norwegian Institute of Bioeconomy Research (NIBIO), N-1431 Ås, Norway; kristin.forfang@nibio.no (K.F.); snorre.hagen@nibio.no (S.H.); 3 Department of Microbiology, Norwegian Veterinary Institute, N-1431 Ås, Norway; marianne.sunde@vetinst.no (M.S.); girum.tessema@vetinst.no (G.T.T.); 4 Division of Biotechnology and Plant Health, Norwegian Institute of Bioeconomy Research (NIBIO), N-1431 Ås, Norway; abdelhameed.elameen@nibio.no (A.E.); marianne.stenrod@nibio.no (M.S.); marit.almvik@nibio.no (M.A.)

**Keywords:** antimicrobials, AMR, ARB, ARG, conventional and organic agriculture, livestock manure, *E. coli*, WGS, quantitative PCR, high-throughput DNA array

## Abstract

The abundance and diversity of antimicrobial-resistant bacteria (ARB) and antimicrobial resistance genes (ARGs) in agricultural landscapes may be important for the spread of antimicrobial resistance (AMR) in the environment. The aim of this study was to apply screening methods for ARB and ARGs to investigate the impact of farming on the prevalence of AMR in a country with low antibiotic usage. We have analyzed samples (n = 644) from soil and wild terrestrial animals and plants (slugs, snails, mice, shrews, earthworms, and red clover) collected over two years in agricultural fields accompanied by nearby control areas with low human activity. All samples were investigated for the occurrence of 35 different ARGs using high-throughput quantitative PCR (HT-qPCR) on a newly developed DNA array. In addition, samples from the first year (n = 415) were investigated with a culture-based approach combined with whole-genome sequencing (WGS) to identify antimicrobial-resistant *E. coli* (AREC). ARGs were detected in 59.5% of all samples (2019 + 2020). AREC, which was only investigated in the 2019 samples, was identified in 1.9% of these. Samples collected in the autumn showed more ARGs and AREC than spring samples, and this was more pronounced for organic fields than for conventional fields. Control areas with low human activity showed lower levels of ARGs and a lack of AREC. The use of livestock manure was correlated with a higher level of ARG load than other farming practices. None of the soil samples contained antibiotics, and no association was found between AMR and the levels of metals or pesticides. High qualitative similarity between HT-qPCR and WGS, together with the positive controls to the validation of our 35 ARG assays, show that the microfluid DNA array may be an efficient screening tool on environmental samples. In conclusion, even in a country with a very low consumption of antimicrobials by production animals, our results support the hypothesis of these animals being a source of AREC and ARGs in agricultural environments, primarily through the use of manure.

## 1. Introduction

Increasing levels of antimicrobial resistance (AMR) and the spread of antimicrobial-resistant bacteria (ARB), as well as antimicrobial resistance genes (ARGs), are considered to be among the most critical global health challenges that we are facing today [1]. The consequences of AMR have been estimated to cause yearly losses of 10 million lives and up to USD 100 trillion of the world’s economy by 2050 [2]. The main driver is our vast consumption of antimicrobials in medicine, agriculture, and aquaculture. Consequently, it is imperative that we address the problem of AMR from a One Health perspective, where the environment may act a reservoir of clinically relevant AMR.

Fecal pollution has been reported to be a major contributor to ARG abundances in the environment [3]. Globally, an estimated 73% of all antimicrobials used have been in livestock production [4,5], and in 2030, the annual antimicrobial consumption by food-producing animals is estimated to pass 200 tons [5]. In livestock, antimicrobials are being used for the treatment and prevention of disease, as well as for growth promotion. Although some regions and countries have banned the use of antimicrobials as growth promoters, high doses for metaphylactic and prophylactic purposes may often still be used [4]. Livestock feces can pollute the environment using manure as fertilizer, or by animal grazing. A number of studies have found enlarged reservoirs of clinically relevant ARB and ARGs in soil after the application of manure from antimicrobial-treated animals, as compared to soils where inorganic fertilizers or no fertilizers had been used (as reviewed by [6]. Furthermore, ARGs have been reported to persist in the soil for more than 120 days [7,8]. In addition, it has been suggested that manure provides an environment that stimulates horizontal gene transfer [9]. The animal consumption of antimicrobials may also contribute to the spread of antimicrobial residues directly to the environment where it can stimulate the evolution and transfer of ARGs. The amount of antimicrobials measured in manure from poultry, swine, and cattle from different countries have been shown to vary from 0.01 to over 100 mg/kg (reviewed by [6]). Tetracyclines, sulfonamides, and fluoroquinolones were the substances most often detected. A study on tetracycline residues in soil fertilized by pig and poultry manure found a direct correlation with the animal consumption of this antimicrobial [10].

Metals like copper, zinc, nickel, mercury, cobalt, and cadmium have also been reported to act as drivers of AMR through cross-resistance or co-resistance [11]. Cross-resistance means that the bacteria develop resistance mechanisms against the metals that are similar to AMR mechanisms, e.g., reduced membrane permeability, expression of efflux pumps, and target site modifications. Co-resistance occurs when ARGs and metal resistance genes are co-located on the same mobile elements, usually on plasmids. ARGs have been observed to occur more frequently in bacteria with rather than without metal resistance genes [12]. The use of pesticides and biocides has also been pin pointed as a potential driver of the development of AMR in the environment through both co- and cross-resistance [13,14]. There are also studies indicating that the combined exposure to pesticides and antimicrobials accelerates the development of AMR, as may be the case in manured soils [15].

ARGs may exist in both bacterial chromosomes and plasmids and as free DNA in the environment, but it is not clear to what extent they may contribute to the spread of AMR among clinically relevant bacteria [16]. ARGs have been documented to date back to 30,000 years ago in permafrost [17], and found in samples from Artic soil [18], and thus their presence in time and environments without modern antimicrobial exposure has been proven. Therefore, differentiating natural AMR in the environment from AMR caused by modern clinical antimicrobial use may be problematic [16,19]. However, recent studies with quantitative PCR show that antimicrobial exposure may lead to highly increased levels in animal manure and soil (reviewed by [20]) and in the estuaries of costal environments [21]. Different methods and instruments for high-throughput qPCR (HT-qPCR) have been applied to efficiently analyze environmental samples for ARGs [22]. Among these technologies for HT-qPCR, the microfluid qPCR technique has been proven to be efficient for the detection of tick-borne pathogens [23], pathogenic bacteria [24], and ARGs [25,26]. We recently developed a set of primers for 35 ARGs to be used in a 96 x microfluid HT-qPCR DNA array [26] and intended for this study to test the reliability and efficiency of the method on environmental and agricultural samples.

In 2020, Norway was reported to be the country with the lowest consumption of antimicrobials by livestock [27]. The aim of the present study was to investigate the impact of farming on the prevalence of ARB and ARGs in the terrestrial environment of agricultural landscapes under the Norwegian low-usage conditions. Areas with limited human activity were used as controls. The samples collected were incubated at 37 °C before further investigations to favor the ARB and ARGs relevant for human and livestock infections. We were also interested in whether season, farming practices, use of livestock manure, and the presence of possible drivers of resistance would influence this prevalence.

## 2. Study Design

**Figure 1:**
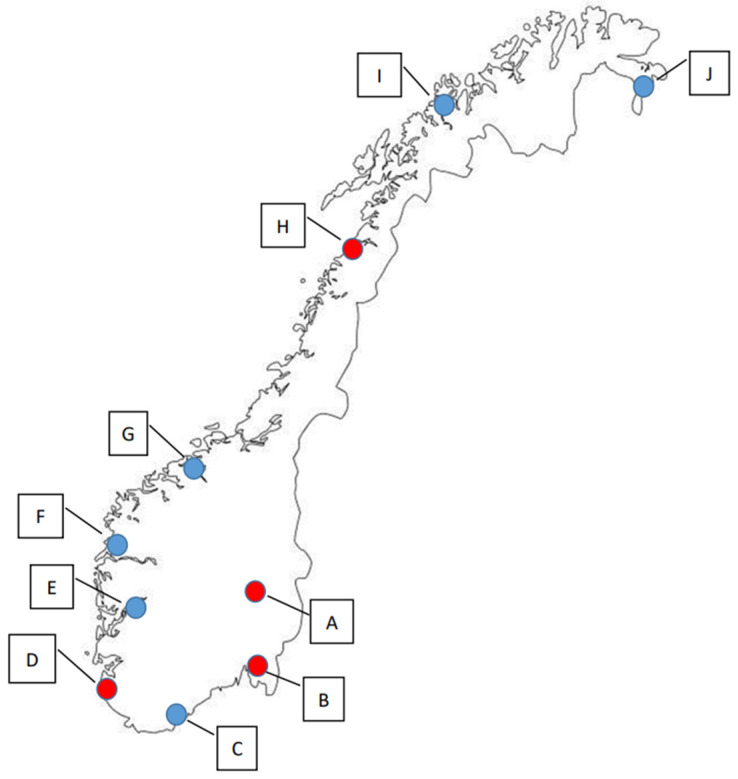
The ten sampling locations in Norway. Red dots (A–D) indicate sampling locations used in both 2019 (spring and autumn) and 2020 (spring), while blue dots (E–J) indicate the locations that were only sampled in the spring of 2020.

In this study, samples were collected from the terrestrial environment of four locations in Norway in 2019, and from ten locations in 2020, which included the four that were also sampled in 2019 (Figure 1, Tables S1–S4). In 2019, sampling was executed both in the spring (n = 197) and the autumn season (n = 218), whereas sampling in 2020 was only performed during the spring season (n = 229).

At each location, samples were from three different area types:

- Conventional, i.e., within or at the edge of a conventional agriculture field;
- Organic, i.e., within or at the edge of an organic agriculture field;
- Control, i.e., in an area without agriculture or settlement, but with natural vegetation (forest, grass, bushes, etc.).

Sample types from the agricultural areas were as follows

- Soil—2019 and 2020;
- Plants (red clover)—2019 and 2020;
- Gastropods (slugs and snails)—2019 and 2020;
- Small mammals (mice and shrews)—2019;
- Earthworms—2019 (only spring).

From the control areas, soil was collected both years. Species were different in the control areas and were therefore not included in the study.

A culture-based approach was used to investigate *E. coli* and AREC from the 2019 samples (n = 415), whereas all samples from both years (n = 644) were investigated for the occurrence of 35 different ARGs using high-throughput quantitative PCR.

Analyses for the presence of antimicrobials and heavy metals were performed on all soil samples collected in the autumn 2019 (n = 12). Analyses for the presence of pesticides were performed on all soil samples both years (n = 84).

## 3. Materials and Methods

**Figure 2:**
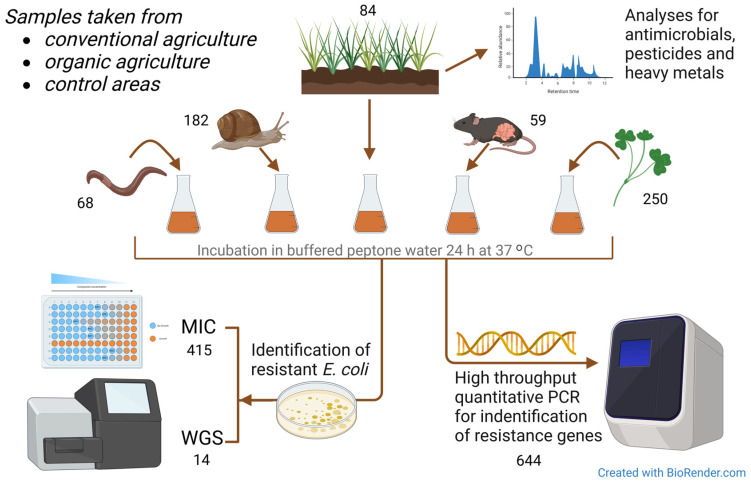
Graphic overview of the materials and methods used in the study. MIC = minimum inhibitory concentration assay for antimicrobial susceptibility testing. WGS = whole-genome sequencing. Numbers are given for each sample type and test procedure.

A graphic display of materials and methods used is given in Figure 2.

### 3.1. Collection and Preparation of Samples

A total of 644 samples were collected, i.e., 84 soil samples, 250 whole red clover plants (*Trifolium pretense*), 182 slugs and snails (including *Arion ater*, *Arion fuscus*, *Arion silvaticus*, *Arion vulgaris*, *Deroceras agreste*, *Deroceras reticulatum*, Helicidae), 59 mice and shrews (including *Apodemus flavicollis*, *Apodemus sylvaticus*, *Microtus agrestis*, *Mus musculus*, *Myodes glareolus*, Soricidae), and 68 earthworms (species not identified).

Norway stretches across very different climatic zones (57–71° N, 4–31° E), and the short spring and autumn seasons are not present in the same weeks and months from south to north and from west to east. Thus, samples were collected in May and June for the spring samples, and August and September for autumn samples, where the sampling dates were adjusted to approximately the same phenological stage of spring and autumn where the red clover, mice, slugs, and earthworms could be sampled for the specific location.

Soil samples (0–5 cm soil depth) were collected from all area types in all locations. In each area, ten individual samples were collected with a soil core borer and pooled before being transported to the laboratory. The water content was determined in all soil samples after drying a subsample overnight at 105 °C. The samples of terrestrial species were collected from the edges of the conventional and organic fields. Mousetraps for small mammals were placed the day before sample collection. On the day of sampling, all samples were individually collected and placed in separate plastic bags before being transported to the laboratory. Samples were kept at cooling temperature until they were processed in the laboratory. The soil samples were split in two; one part to test for AREC and ARGs, and the other to test for possible drivers of AMR. To investigate the samples for the presence of AREC and ARGs, red clover, slugs, and earthworms were cut into pieces. For red clover, the whole plant above ground was sampled (the roots were not included). From the mice, only the intestines were sampled, and from the soil, 25 g was used. Each sample was weighed, mixed with buffered peptone water (BPW) 1:10 *w*/*v*, and mixed either manually or in a stomacher. After 24 h of incubation at 37 ± 1 °C, 1 mL from each sample was frozen at −70 °C for DNA analyses. In 2019, 10 µL from each sample was also spread on MacConkey agar plates (Difco), which were incubated overnight at 37 ± 1 °C before further assessment.

### 3.2. Isolation and Identification of E. coli

*E. coli* was chosen as the bacterial indicator species. From the overnight MacConkey agar plates, colonies were identified by matrix-assisted laser desorption/ionization time-of-flight mass spectrometer (MALDI-TOF MS, Bruker Daltonics GmbH, Billerica, Massachusetts, USA. Where present, three *E. coli* colonies from each sample were stored at −80 °C to be used in further investigations.

### 3.3. Susceptibility Testing of E. coli

All *E. coli* isolates were subjected to minimum inhibitory concentration (MIC) determination by using the same quality-controlled broth microdilution method and the same EUVSEC panel from Sensititre^®^ TREK (Trek Diagnostic System Ltd., United Kingdom as the Norwegian NORM-VET monitoring program for antimicrobial resistance in animals, food, and feed [28]. Consequently, each bacterial isolate was tested against 14 antimicrobial agents to which *E. coli* is naturally susceptible (Table S5). In addition, all *E. coli* isolates with a positive qPCR DNA chip signal for the genes *strA* (*aph(3″)-Ib*) and *strB* (*aph(6)-Id*) were tested for phenotypic resistance to streptomycin using ETEST^®^ (bioMérieux, Marcy-l’Étoile, France). Epidemiological cut-off values (ECOFF) recommended by the European Committee on Antimicrobial Susceptibility Testing (EUCAST, https://www.eucast.org/, accessed 10 October 2020) were used to define the bacteria as wild-type (susceptible) or non-wild-type (resistant). An *E. coli* isolate displaying phenotypic resistance to one or more antimicrobials included in these tests was defined as antimicrobial-resistant *E. coli* (AREC).

### 3.4. Whole-Genome Sequencing of Antimicrobial-Resistant E. coli (AREC)

DNA from each isolate was extracted using the QIAamp^®^ DNA Mini Kit (Qiagen, Hilden, Germany) according to the manufacturer’s protocol, with some minor modifications. The optional RNase A step was included, and 100 μL 10 mM Tris, pH 8, was used as elution buffer. The purity of the DNA extracts was determined using MySpec (VWR^®^, Radnor, Pennsylvania, USA) and DNA concentrations were determined using the Tecan Spark Fluorometer (Tecan Trading AG, Männedorf, Switzerland) with the Qubit broad range kit. The DNA was prepared using the Nextera™ DNA Flex library preparation kit (Illumina DNA Prep, Illumina, Inc., San Diego, California, USA) and sequenced on an Illumina MiSeq resulting in 300 bp paired-end reads.

Analyses were performed using the Bifrost pipeline (https://zenodo.org/records/4043861, accessed 18 January 2021). Quality control was performed using FastQC v0.11.9 (Babraham Bioinformatics. FastQC, available at http://www.bioinformatics.babraham.ac.uk/projects/fastqc/ (accessed 18 January 2021), and MultiQC v1.9 [29] to collate the FastQC data. Genomes were assembled using Trimmomatic v0.39 [30] and SPAdes v3.14.0 [31] using parameters with coverage cutoff set to “auto” and “--careful” settings and excluding contigs shorter than 500 nucleotides. The assemblies were polished by running Pilon v1.23 [32] before genome annotation with Prokka v1.14.5 [33]. QUAST v5.0.2 [34] was used for evaluating the assemblies.

Multi-locus sequence typing (MLST) was performed using ARIBA [35] using the scheme hosted by Enterobase [36]. Blast v2.11.0 was run to search for class 1 and 2 integrase. ResFinder v4.1 on the Centre for Genomic Epidemiology (CGE) website (https://www.genomicepidemiology.org/services/, accessed 9 November 2021) was used to detect acquired genes and point mutations causing AMR [37,38,39]. SpeciesFinder v2.0 and KmerFinder v3.2 at the CGE website were used for species confirmation.

To investigate the clonality of the thirteen *E. coli* isolates, the pipeline A Tool for Prokaryotic Phylogeny and Clustering Analysis (ALPPACA) v2.3.1 was run on the assemblies using the ‘core gene’ parameter [40]. An IQTree generated was visualized using the interactive Tree of Life (iTOL) v 6.9 [41].

### 3.5. High-Throughput Quantitative PCR (HT-qPCR)

DNA was extracted from 500 µL of each BPW sample from the overnight cultures of material from red clover, slugs, mice intestine, and earthworms using the DNeasy Blood & Tissue kit (Qiagen) with an optimized protocol for Gram-negative bacteria, as described by the manufacturer. Prior to DNA extraction, the 500 µL BPW cultures were centrifuged for 10 min at 5000× *g* followed by the resuspension of the pellet in 180 µL ATL-buffer (Qiagen). For the soil samples, 500 µL of the incubated BPW cultures were centrifuged at 10,000× *g* for 10 min followed by removing 400 µL of the supernatant. The pellet in 100 µL remaining liquid was used for DNA extraction applying the DNeasy Power Soil kit (Qiagen) according to the manufacturer’s instructions. All DNA samples were eluted in a total volume of 100 µL elution solution.

The HT-qPCR DNA analysis was performed using a setup with the BioMark HD system with the 96.96 Dynamic Array Integrated fluid circuits (ICFs) for real-time PCR (Fluidigm, San Francisco, CA, USA) [26]. The presence of ARGs in the samples were determined using 46 assays developed to detect 35 ARGs [26] responsible for resistance to ten antimicrobial classes, including beta-lactams, tetracyclines, aminoglycosides, amphenicols, fluoroquinolones, sulfonamides, dihydrofolate reductase (DHFR) inhibitors, glycopeptides, colistin, and macrolide–lincosamide–streptogramin B (MLS). The ARGs with their respective antimicrobials are presented in Table S6. The qPCR DNA Array contained 44 assays for ARGs, an assay for the detection of microbial DNA (16S rRNA gene, referred to as “16S” hereafter) and an assay for the class 1 integron–integrase gene *intl1*. Among the 46 assays, we used a single primer set for 29 genes, with two primer sets for eight genes and three primer sets for three genes (the complete list of the primer sets is presented in Røken et al. [26]). Pre-amplification was performed with 1.25 µL of eluted DNA and a final primer concentration of 0.05 µM in a 14-cycled specific target amplification step due to the low volume (6.7 nL) in each reaction chamber of the qPCR-array. The pre-amplification conditions were as follows: Initial denaturation at 95 °C for 15 min, 14 cycles at 95 °C for 15 s, and 60 °C for 4 min. Eleven positive controls with confirmed presence of different ARGs and four negative controls were included in each run. The 96.96 DNA Array IFC (Fluidigm, USA) was primed and loaded with pre-amplified DNA (2.25 µL) and EvaGreen assays (Invitrogen, Waltham, MA, USA) in two replicates according to the manufacturer’s protocol. Initially, the samples underwent thermal mixing at 70 °C for 40 min followed by 60 °C for 30 s, followed by a hot start at 98 °C for 2 min, 40 cycles at 98 °C for 5 s, and 60 °C for 20 s, ending with a melting curve analysis at 60 °C for 3 s followed by a 1 °C/3 s increase to 95 °C. The standard curves of the 11 positive controls were tested against all 46 assays to determine the slopes and intercept for the quantification of each assay, among which 32 assays had a confirmed presence of the given ARGs, while the remaining assays lacked detection in the positive controls. Technical data and standard curves are presented in Figure S1 and Table S14. Data collection was performed using the Biomark HD Data Collection software (Fluidigm). The positive controls were used to correct the CT value before quantification. The quantification of the ARGs present was conducted in the Fluidigm Real-Time PCR Analysis software (version 4.5.2) using Equation (1), in which the CT value represented the mean of the duplicates.

(1) $$ARGs\left(\frac{ng}{\mu L}\right)=10^{\frac{\left(CT+{CT}_{corr}\right)-intercept}{slope}}$$

### 3.6. Testing for Antimicrobial Agents and Pesticides in Soil

The soil samples (0–5 cm soil depth) were stored frozen (−20 °C) after arriving at the laboratory. The water content was determined in all soil samples after drying a subsample overnight at 105 °C. For antimicrobial analysis, 10 g of fresh soil samples were extracted with acetonitrile. A complementary screening database with 100 antimicrobials was used in the analysis of the samples, based on the Thermo Fisher Scientific Environmental and Food Safety (EFS) HRAM Compound Database and Spectral Library (https://www.thermofisher.com/order/catalog/product/IQLAAEGABSFAPYMBHL, 2016 version, expanded with in-house reference compounds, accessed on 27 August 2024) and used with Tracefinder 4.1 and mzVault 2.1 software. The samples were analyzed using high-resolution mass spectrometry (HRMS) Q-Orbitrap instrument in full MS-data-dependent MS2 (FullMS-ddMS2) mode with searches directed towards a targeted in-house database for 450 pesticides and metabolites in soil and a non-targeted database for 108 veterinary drugs. The databases were based on the Thermo Fisher Scientific Environmental and Food safety (EFS) HRAM Compound Database and Spectral Library (https://www.thermofisher.com/order/catalog/product/IQLAAEGABSFAPYMBHL, accessed on 27 August 2024) and used with the Tracefinder 4.1 and mzVault 2.1 software. Samples were analyzed on a UHPLC-Q Exactive Orbitrap-HRMS system (Thermo Fisher Scientific™, Bremen, Germany) composed of a Dionex Ultimate 3000 liquid chromatograph equipped with an LPG-3400XRS pump. The samples (injection volume 1 µL) were introduced into the HRMS after heated electrospray ionization (HESI). Chromatographic separation was achieved on a Thermo Accucore aQ column (100 × 2.1 mm, 2.6 μm) with a gradient of 5 mM NH_4_COOH/0.1% NH4Formiate in water (A) or in methanol (B), respectively. The flow rate was 300 μL/min. The total runtime was 18 min. The Orbitrap was used in (+) HESI full scan mode at a resolution of 70,000 FWHM, AGC target 1E6, maximum injection time (IT) of 50 ms, and scan range of 100–1100 *m*/*z*. Soil samples were first screened in the FMS-DIA mode, succeeded by the targeted FMS-ddMS2 mode with the inclusion list. The identification criteria were retention time (RT) matched to reference standard, precursor ion accurate *m*/*z* mass within 5 ppm accuracy, and the presence of at least one targeted production with accurate mass within 5 ppm accuracy and produced by the fragmentation of the precursor ion. An in-house spectra library of product ion spectra (MS2) for the compounds aided in the identification. Quantification was based on the peak area of the precursor ions. Calibration standards were prepared in the range of 1–200 ng/mL in vial, corresponding to 0.005–1 µg/L water, or 1–200 µg/kg soil. Limits of quantification (LOQs) for pesticides and metabolites were typically between 1 and 10 µg/kg in soil.

### 3.7. Testing for Metals in Soil

Soil samples were dried in a drying cabinet (25 °C) with forced air circulation, sieved (2 mm), and grinded in a mortar. The sample (0.2 g) was decomposed in 4 mL 65% nitric acid in an Ultrawave microwave oven, diluted with 20 mL deionized water, and analyzed with inductively coupled plasma–optical emission spectrometry (ICP-OES) on an iCAP 6000 series IPC Emission spectrometer (Thermo Fisher Scientific, Cambridge, UK) for the determination of the amounts of elements (i.e., metals and others).

### 3.8. Statistical Analyses

The quantification of each ARG (ARG value) in a sample was given as a relative concentration against 16S (ng/µL). Sum ARG was calculated by summarizing the ARG values of detected ARGs (ARG/16S) for each sample. ARG Score was calculated for each gene by summarizing the gene’s ARG value (one assay pr. gene) in all samples. Statistical analysis was conducted in Excel^®^ 2016 and the software R version 4.3.2 [42]. *p*-values ≤ 0.05 were considered statistically significant.

## 4. Results

### 4.1. Isolation of E. coli

In total, 111 (26.7%) samples with a total of 313 *E. coli* isolates were identified in 2019 (Table S7). Large variations were observed between sample types (Table 1). Furthermore, significant differences in the prevalence of *E. coli*-positive samples were observed within each of the variables Location, Season, and Area type. The prevalence was higher in in location D than in the three other locations (*p* < 0.01, 39.7% vs. 19.0–22.2%, respectively), higher in the autumn than in the spring (*p* < 0.01, 36.7% vs. 15.7%, respectively), and higher in organic areas than in conventional areas (*p* = 0.03, 31.6% vs. 20.7%, respectively). Earthworms, which displayed a much lower prevalence of *E. coli*-positive samples than the other sample types, were only collected during the spring season. However, when comparison between seasons was performed without the earthworm samples included, the difference was still statistically significant (*p* = 0.03, 36.7% vs. 23.3%).

### 4.2. Antimicrobial-Resistant E. coli (AREC)

We identified eight samples with *E. coli* that displayed phenotypic resistance, i.e., 1.9% of all samples and 7.2% of the *E. coli*-positive samples in 2019 (Table 2). The positive samples came from three of the four locations (Tables S9 and S10). Seven were autumn samples and one was a spring sample. Furthermore, seven of the samples were from organic areas, one was from a conventional area, and none were from control areas.

Phenotypic resistance to ampicillin and chloramphenicol were most observed, followed by resistance to streptomycin, sulfamethoxazole, and tetracycline (Table S8). A total of 18 phenotypically resistant isolates were obtained from these eight samples. The WGS of 14 of these isolates confirmed that all were *E. coli*, and that all but one (3920-11-3) carried genes corresponding to the observed phenotypic resistance (Table 2). Resistant isolates from the same samples displayed the same resistance patterns. Two isolates from one of the samples (3820-1) had the same sequence type, and carried the genes *sul2*, *aph(3″)-lb* (*strA*), and *aph(6)-ld* (*strB*) without expressing resistance to sulfamethoxazole or streptomycin in the phenotype tests. Furthermore, all resistant isolates from the organic area of location A displayed the same resistance pattern. Of those subjected to WGS, all had the same sequence type. Given the resolution of the analysis, the results indicate that these isolates were closely related.

### 4.3. Antimicrobial Resistance Genes (ARGs) in Agricultural and Control Landscapes in Norway

From 644 samples, 638 samples were positive for 16S, and 383 samples (59.5%) were found to be above the detection threshold for one or several of the 35 different ARGs (Table 3, Tables S6, S15 and S16). Only six samples (0.9%) were negative for 16S (Table 3), which may represent samples with bacterial DNA below the detection threshold, degraded DNA, or contamination by inhibitors of PCR in the sample. 

The ARG Score for each gene varied between 0.0 and 38.1 (Table 4). The highest ARG Scores were observed for genes conferring resistance against betalactams, tetracyclines, streptomycin, sulfonamides, and chloramphenicol. High ARG Scores were also found for the gene *intl1* encoding class 1 integron–integrase, and *oqxAB* encoding a plasmid-borne multidrug efflux pump.

**Figure 3:**
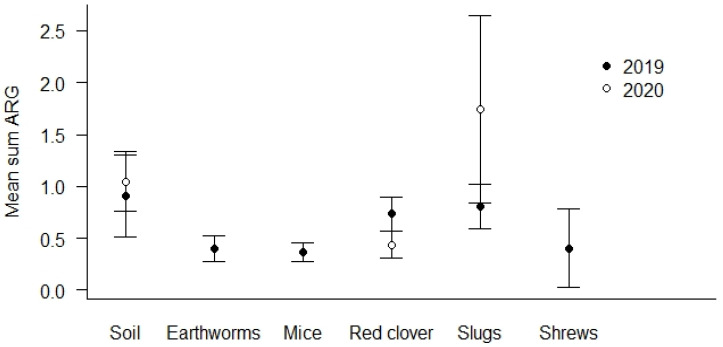
Mean Sum ARG for sample types. Values are based on the HT-qPCR detection of 35 ARGs on a Biomark Fluidigm 96.96 Dynamic DNA array. All ARG values were normalized with respect to 16S. Vertical bars represent ± standard error. Soil and slug samples with the highest mean Sum ARG were statistically significantly higher relative to red clover in spring samples of 2020 (*p* < 0.01).

The highest prevalence of ARG positive samples was found in the soil samples (69.0%, see Table 4). The lowest number of ARG positive samples was detected in earthworms (44.1%). Soil and slug samples show the overall highest mean Sum ARG, but this was only statistically significant for soil and slugs relative to red clover in the spring samples from 2020 (*p* < 0.01, Figure 3).

**Figure 4:**
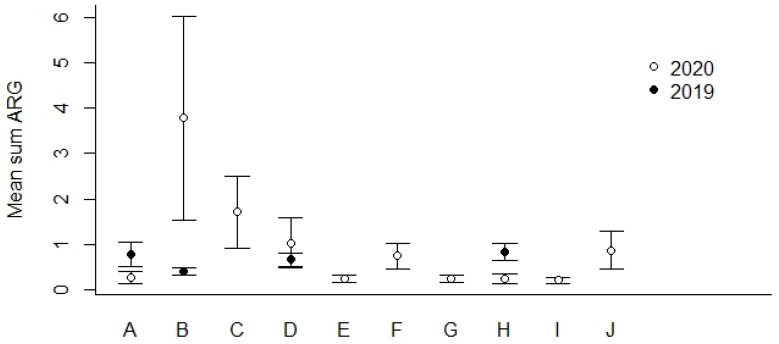
Mean Sum ARG for sampling locations A–J in 2019 and 2020 (see Figure 1 for geographical location and Table 5 for the number of ARG-positive samples). Values are based on the HT-qPCR detection of 35 ARGs on a Biomark Fluidigm96.96 Dynamic DNA array. All ARG values were normalized with respect to 16S. Vertical bars represent ± standard error.

We found that the geographical locations B and C (Figure 1) showed the highest percentages of ARG-positive samples (Table 5). In general, we detected lower percentages of ARG-positive samples among the most northern sampling locations (F–J) compared to locations more to the south in Norway (A–E). The mean Sum ARG was below 1 for all sampling locations except for B and C collected in the year of 2020 (Figure 4).

**Figure 5:**
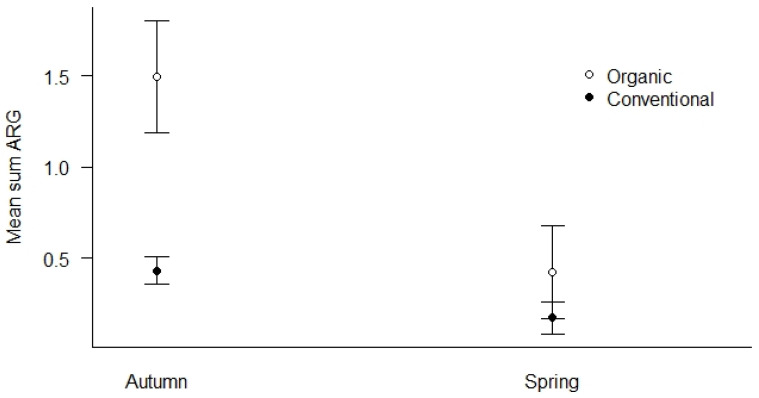
Mean Sum ARG for the season (spring vs. autumn) and type of agricultural practices (conventional vs. organic) for all samples in 2019. Values are based on HT-qPCR detection of 35 ARGs on a Biomark Fluidigm 96.96 Dynamic DNA array. All ARG values were normalized with respect to 16S. Vertical bars represent ± standard error. Organic fields show significantly elevated levels of ARGs compared to conventional fields in the autumn (*p* < 0.01). Both agricultural practices show higher levels of ARGs in autumn relative to spring with the most pronounced elevation in autumn for organic fields (*p* < 0.01).

We used all samples collected in both spring and autumn of 2019 to investigate how season and agricultural practices influenced the mean Sum ARG of the samples (Figure 5). First, we found that organic fields had elevated levels of mean Sum ARG compared to conventional fields in autumn (*p* < 0.01), while the difference was less pronounced in spring (Figure 5). Second, both agricultural practices show higher levels of mean Sum ARG in autumn relative to spring, with the most pronounced elevation for organic fields (*p* < 0.01).

**Figure 6:**
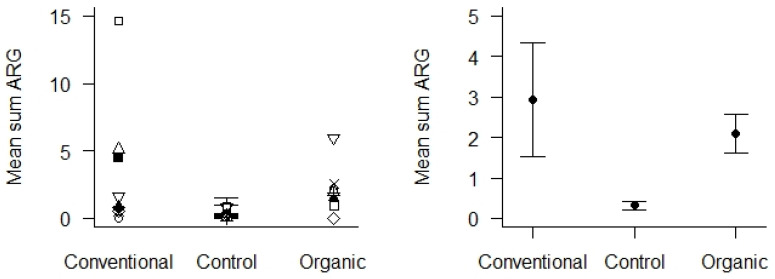
Mean Sum ARG in soil samples from 2020 with respect to the type of agricultural practices (conventional vs. organic) and control areas. Values are based on the HT-qPCR detection of 35 ARGs on a Biomark Fluidigm 96.96 Dynamic DNA array. All ARG values were normalized with respect to 16S. **Left panel**: Mean Sum ARG for each of the 10 locations plotted for the two types of agricultural practices and the control areas. **Right panel**: Mean Sum ARG with standard error (vertical bars) was significantly lower for soil samples from control areas relative to soil samples from agricultural areas (*p* < 0.01).

Control samples were soil from areas with absent or low human or livestock activity located in the same geographical area as the sampled agricultural areas. These were compared with soil samples from the agricultural areas, using only samples from 2020 to obtain enough comparable control samples. We found that the mean Sum ARG in soil samples from the control areas was very low (Figure 6), and significantly lower than the soil samples from the agricultural areas (*p* < 0.01). We could not detect any differences in Sum ARG between the conventional and organic fields with respect to the soil samples collected in 2020.

**Figure 7:**
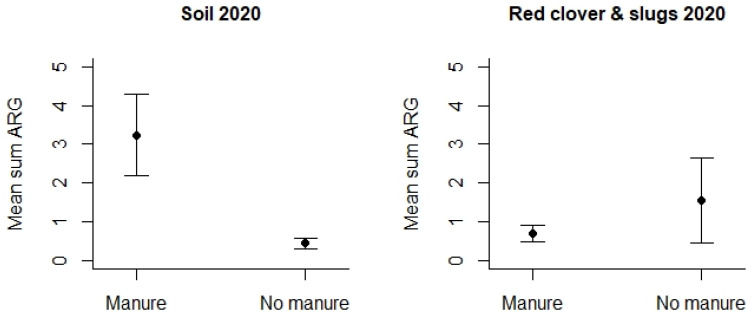
Mean Sum ARG in soil samples and other samples from 2020 with respect to the type of use of animal manure versus other fertilizers. Values are based on the HT-qPCR detection of 35 ARGs on a Biomark Fluidigm 96.96 Dynamic DNA array. All ARG values were normalized with respect to 16S. Vertical bars represent ± standard error. Mean Sum ARG was significantly lower for soil samples from agricultural fields without animal manure (control areas) relative to soil samples from agricultural areas where animal manure had been applied (*p* < 0.01).

However, turning to the use of animal manure, this was studied by means of samples collected in 2020 (Table S4). We detected that agricultural fields where manure had been applied displayed significantly higher mean Sum ARG in the soil samples compared to soil samples from the fields where no fertilizer or other types of fertilizers had been used (*p* < 0.01 see Figure 7). This difference could only be detected for soil samples, and any differences related to the use of manure were lacking for the other samples, i.e., slugs/snails and red clover.

### 4.4. Comparison of E. coli WGS and ARG Identification Using HT-qPCR

Twelve of the *E. coli* isolates that displayed a presence of ARGs when subjected to WGS were also tested in the HT-qPCR. All genes identified by WGS displayed high ARG values in the HT-qPCR of the respective isolates, and that of the samples the isolates originated from (Table S11). In addition, strong HT-qPCR signals from other genes were identified in two of the samples, and weak signals from the other genes in three of the samples. The detection of these genes may have been caused by other bacteria than the isolated *E. coli* present in the samples.

### 4.5. Antimicrobials, Heavy Metals, and Pesticides in Soil

We analyzed all soil samples collected in autumn 2019 (n = 12) for the presence of antimicrobials and heavy metals. None of the antimicrobial agents we analyzed for were found in any of the samples. Furthermore, the levels of ARGs in any of the four locations or the type of field (conventional, organic, or control) could not be explained by variations in the concentrations of heavy metals in the soil (Table S12). Analyses for the presence of pesticides were performed on all soil samples from both years. With one exception, pesticides were only detected in the soil from areas with conventional farming (Table S13A–C). The exception was a soil sample from an organic area at location A in 2020, which showed residues of pesticides (sum pesticides detected 50.7 µg kg^−1^).

No statistically significant results were found for the correlation analysis of mean sum ARG and mean sum pesticides for the conventional fields. Furthermore, due to the few detections of each pesticide, the statistical analysis of any connections between the residues of individual pesticides and the detections of ARGs were not possible and would require a substantially more elaborate sampling scheme.

## 5. Discussion

In the present study, the prevalence of AMR in the Norwegian terrestrial environment was studied by identifying antimicrobial-resistant *E. coli* (AREC) indicator bacteria, as well as antimicrobial resistance genes (ARGs), in samples from agricultural landscapes and control areas at the same locations with low human activity. AREC was identified in 1.9% of 415 investigated samples, and ARGs were identified in 59.5% of all samples (n = 644). The mean Sum ARG, representing the ARG load, was significantly higher in areas with agricultural landscapes than in the control areas. Likewise, the prevalence of AREC was also higher in these landscapes, but statistical calculations were not performed due to the low number of AREC isolates. This is in agreement with a study in Great Britain, which also found a significantly higher level of ARB in agricultural land compared to urban or semi-natural sites [43]. In the present study, autumn samples displayed a higher ARG load than the spring samples. This was also the case for AREC. However, the percentage of samples positive for *E. coli* was also higher in the autumn than in the spring. This indicates that *E. coli* and AREC were added to during the production season through fecal pollution, and possibly that the warmer conditions stimulated bacterial growth, evolution, and gene transfer. At one sampling area (organic area at location A), four autumn samples (three separate plants and one slug) all harbored similar *E. coli* isolates, most likely due to pollution from a common source.

The AREC isolates identified displayed phenotypic and genotypic resistance to ampicillin, sulfamethoxazole, tetracycline, chloramphenicol, and streptomycin. The ARG Scores in the HT-qPCR array also showed that genes conferring resistance to these antimicrobials were the most abundant. *E. coli* indicator bacteria resistant to the same antimicrobials were also isolated from Norwegian farm animals like cows, pigs, and broilers, as well as leafy greens and herbs in the Norwegian NORM-VET monitoring program for antimicrobial resistance in animals, food, and feed (NORM VET 2019). Samples collected in the autumn season of 2019 in the present study displayed a higher prevalence of both *E. coli* and AREC, as well as a higher mean Sum ARG in organic areas than in conventional areas. Similar findings were recently reported from vegetable farms in the Jiangsu province of China, where a higher abundance of ARGs was detected in soil samples from the organic farms than the conventional ones [44]. In our study, the organic areas primarily used cattle manure as fertilizer. Accordingly, in soil samples collected in 2020, the presence of ARGs correlated with the use of manure. A similar correlation with the other sample types was not observed in 2020, but this may have been due to samples in 2020 only being collected during the spring season. The results might therefore suggest that AREC and ARGs spread with manure as field fertilizer may need some time to contaminate the fauna and the flora of the field edge-zones. In Norway, antimicrobials are only used in relatively small amounts for the treatment of individual production animals, because the use of antimicrobials as prophylactics or growth promoters is forbidden. In 2019, approximately 90% the antimicrobials used for cattle were beta-lactamase-sensitive penicillin, in addition to smaller amounts of other penicillin, aminoglycosides, tetracyclines, and sulfonamides in combination with trimethoprim [28], thus matching the resistance observed in the animals and the agricultural environment. The use of antimicrobials in farm animals has previously been identified as a risk factor for the spread and development of ARB in the environment [6]. In addition, previous studies with HT-qPCR have documented positive correlations between multiple genes and concentrations of antimicrobials in manures and compost in China [45]. Although we observed a low background of ARGs in agricultural landscapes in Norway, we did find that the elevation of ARGs was correlated with the use of animal manure in soil from farmlands. Earlier studies showed that soil is a natural reservoir for ARGs, and that animal manure increases soil ARGs [20], which fits well with the results of our study.

Consequently, all our results support the hypothesis of production animals being a source of AREC and ARGs in agricultural environments primarily through the use of manure. Interestingly, an earlier survey of red foxes in Norway showed that these animals also harbored *E. coli* resistant to mainly the same antimicrobials, and the occurrence of AREC was associated with human population density [46]. Likewise, the same AREC was found in Norwegian roe deer, a species which often chooses habitats close to human activity [47]. However, whether these animals have acquired these types of AREC in the agricultural environments or other places with human activity is not known.

In the present study, the samples were incubated at 37 °C before further investigations to favor the chosen indicator bacterium, and the ARGs carried by this, as well as by the other species relevant for infections in humans and livestock. We used *E. coli* as an indicator of AMR, as this species is widely used and recommended for environmental studies as a marker for fecal contamination [48]. This allows our results to be compared with those of other relevant studies. However, the throughput of this method was relatively low, only one bacterial species was studied, and this was isolated from 1–80% of the samples, depending on sample type. A comparison of the results from the WGS and the HT-qPCR on resistant *E. coli* isolated in the present study displayed high conformity, as all ARGs identified by WGS were also recognized by HT-qPCR. Furthermore, the HT-qPCR of the samples from which these resistant *E. coli* were isolated also identified the same ARGs. The higher prevalence of samples with ARGs than those of AREC may indicate that free DNA and/or other resistant bacteria were present in the samples where *E. coli* was not isolated. The HT-qPCR array enabled a larger number of samples to be studied, and the ARGs detected were not restricted to one bacterial species. The method quantified both the ARG load of each sample (i.e., Sum ARG), as well as the load of each ARG in the material (i.e., ARG Score) which was relevant for human infections.

The HT-qPCR detection of ARG measures the amount-specific ARG DNA relative to the amount of the 16S RNA gene DNA. The microfluid technique used in the present study has a very high-throughput capacity compared to other HT-qPCR methods [22], but the low sample input may to some degree negatively affect the sensitivity level of the 35 assays. However, the lowest sensitivity levels may not be of major interest for the study as we aim to discover an AMR pollution that has clinical relevance. Our HT-qPCR PCR primers are selected from previous work by others (see [26]), and are typically designed as consensus primers that will not differentiate specific gene subtypes, as exemplified by the blaTEM and blaSHV genes where further sequence analyses are required to determine the exact gene subtype [49]. Moreover, compared to metagenomic approaches, the HT-qPCR has better sensitivity, lower cost pr. sample, and does not involve time-consuming bioinformatics [22]. Based on the efficiency of the PCR assays and the chosen qPCR method, the different studies on ARGs in the environment may not be directly comparable [20]. In addition, the reliance on 16S copy number (i.e., between 1 and 15 copies for different species) for normalization may be a bias between different environmental samples and may only be adjusted for by the genomic sequencing of the sample [50]. The 16S copy numbers may also influence the quantitative measures of our study, but we find high qualitative similarity between HT-qPCR and WGS which, together with the positive controls, contributed to the validation of our 35 ARGs on the Biomark Fludigm 96.96 DNA array. Future studies may also include more positive controls and additional normalization genes.

Based on our experience from the present study, we suggest that the described HT-qPCR be the preferred method for the environmental surveillance of AMR. The method can be easily combined with the isolation of the indicator bacteria from ARG-positive samples for additional information such as the presence of multi-resistant bacteria and phylogenetic relationships between the resistant bacteria.

A weakness of this study, which primarily affected the AREC investigation, was related to the choice of sample types. In 2019, several sample types were included, i.e., soil, plants, and various small terrestrial animals. However, it turned out to be difficult to find the planned number of each sample type in all areas and locations. In addition, the prevalence of the indicator bacterium *E. coli* displayed large variations between the sample types, from 1.5% in earthworms to 79.7% in mice/shrews. This may have affected the observed differences in the probability of finding *E. coli,* and thereby AREC, between the locations and area types. In the autumn, almost three times as many mice/shrews were sampled from the conventional areas than from the organic, and the difference in AREC prevalence between the two area types may therefore have been even larger than observed. This uncertainty was less relevant for the ARG results, as the difference between the sample types was much smaller. Considering this limitation, our study showed that soil together with slugs/snails and red clover are good candidates for future AMR surveillance in terrestrial environments, combining easy collection at most locations with an acceptable prevalence of the indicator bacteria. In this study, each soil sample consisted of ten smaller samples from the same area that were pooled before investigation, whereas all other sample types were from separate individuals (mammals, gastropod, earthworms, and plants). Consequently, the AMR prevalence in a soil sample may not be directly comparable with that of an individual sample. However, such comparisons were not relevant as we aimed to measure the AMR load of the different areas, not to perform any statistical studies within an area. Our focus on bacteria with relevance to humans and animals (i.e., preincubation at 37 °C) may underestimate other environmental bacteria that may also carry ARGs. ARGs are also expected to be natural since ancient times [17]. Environmental microorganisms in the soil are the source of both antibiotics and the correspondent resistance [20], and thus may also represent a potential future for clinically relevant AMR after HGT of ARGs. In this study, we have focused on screening for the most clinically relevant AMR, but the HT-qPCR array method may be applied in future studies for screening other environmental bacteria for ARGs.

In this project, the concentrations of the potential drivers related to agriculture, i.e., antimicrobials, heavy metals, and pesticides, were measured in soil samples for comparison with the levels of AMR in the same areas. It is not a surprise that antimicrobials were not detected, as the usage in Norwegian farm animals is very low. The concentrations of metals in the soil at the different locations were too similar to explain any of the AMR differences observed in this material. The temporal and spatial variability in soil pesticide concentrations evident from the sample dataset suggests the need for a more comprehensive sampling scheme to elucidate any connections between pesticides and AMR in soil. 

In conclusion, our results indicate that even in a country like Norway, with one of the world’s lowest levels of antimicrobial consumption by farm animals, manure from these animals still may be a source of ARB and ARGs in agricultural environments. The study also shows that the HT-qPCR array used is an efficient screening tool. Additional information from the characterization of indicator bacteria may be obtained by the isolation of these from positive ARG samples.

## Figures and Tables

**Table 1:** Isolation of *E. coli* from different sample types collected in 2019. CI = confidence interval.

Sample Type	Total No of Samples	*E. coli*-Positive Samples		
		No	%	95% CI
Soil	24	17	70.8	48.9–87.4
Red clover	150	26	17.3	11.6–24.4
Slug/snail	114	20	17.5	11.1–25.8
Mouse/shrew	59	47	79.7	67.2–89.0
Earthworm *	68	1	1.5	0.04–7.9
**Sum**	**415**	**111**	**26.7**	**22.5–31.3**

* only collected in the spring.

**Table 2:** Samples collected in 2019 with *E. coli* isolates displaying phenotypic resistance. Results for all three isolates from each sample are included. For isolates that were subjected to whole-genome sequencing (WGS), the sequence type (ST) and identified antimicrobial resistance genes (ARGs) are listed. The genes confer resistance to the following antimicrobials: *bla_TEM_*—beta-lactamases, *cat*—phenicoles, *flo*—phenicoles, *sul*—sulfonamides, *tet*—tetracyclines, *aph(3′)* and *aph(6″)*, i.e., *strA* and *strB*,—streptomycin. Unless noted, the genes displayed both coverage and identity of 100%.

Location Area Season	Sample		Isolate No	Antimicrobials to Which Each Isolate Displays Phenotypic Resistance	ST	ARGs Identified by WGS						
	Type	ID				*bla_TEM_*	*cat*	*flo*	*sul*	*tet*	*aph(3″)*	*aph(6)*
D Organic spring	Slug	1860-20	1	None	-							
			2	Ampicillin, sulfamethoxazole, tetracycline, streptomycin	69	1C			2	A	Ib	Id *
			3	None	-							
A Organic Autumn	Red clover	3544-3	1	Ampicillin, chloramphenicol	362	1B	A1 *					
			2	Ampicillin, chloramphenicol	362	1B	A1 *					
			3	Ampicillin, chloramphenicol	-							
	Red clover	3544-5	1	Ampicillin, chloramphenicol	362	1B	A1 *					
			2	Ampicillin, chloramphenicol	362	1B	A1 *					
			3	Ampicillin, chloramphenicol	-							
	Red clover	3544-9	1	Ampicillin, chloramphenicol	362	1B	A1 *					
			2	Ampicillin, chloramphenicol	362	1B	A1 *					
			3	Ampicillin, chloramphenicol	-							
	Slug	3544-20	1	Ampicillin, chloramphenicol	362	1B	A1 *					
			2	Ampicillin, chloramphenicol	362	1B	A1 *					
			3	Ampicillin, chloramphenicol	-							
B Conventional autumn	Mouse	3942-4	1	None	-							
			2	None	-							
			3	Ampicillin	1157	1D						
D Organic autumn	Soil	3820-1	1	Chloramphenicol	2522			R **	2		Ib *	Id
			2	Chloramphenicol	2522			R **	2		Ib *	Id
			3	None	-							
	Red clover	3820-11	1	Streptomycin	549						Ib *	Id
			2	None	-							
			3	Ampicillin	1170							

- WGS was not performed; * gene with coverage = 100%, but identity < 100%; ** gene with coverage < 100% and identity < 100%.

**Table 3:** ARGs in different sample types from ten geographical locations in Norway during 2019 and 2020 based on the HT-qPCR detection of 35 ARGs on a Biomark Fluidigm 96.96 Dynamic DNA array. All ARG values were normalized with respect to 16S and regarded as positive above the set detection threshold.

Sample Type	No. of Samples			% ARG Positive
	Total No	16S Positive	ARG Positive	
**Soil**	84	80	58	69.0
**Earthworm**	68	66	30	44.1
**Mouse/shrew**	59	59	36	61.0
**Red clover**	250	250	147	58.8
**Slug/snail**	183	183	111	60.7
**All**	**644**	**638**	**383**	**59.5**

**Table 4:** ARG Score for each of the 35 genes investigated in samples from 2019 (n = 415) and 2020 (n = 229), as calculated by summarizing each gene’s ARG value in the HT-qPCR for all samples each year (strA = *aph(3″)*, strB = *aph(6)*).

Gene	ARG Score		Gene	ARG Score		Gene	ARG Score	
	2019	2020		2019	2020		2019	2020
***aac3***	0.190	2.647	***catA***	21.064	0.000	***qnrA1***	0.023	0.012
***aac6***	9.516	1.318	***cmlA***	0.000	0.000	***qnrB1***	1.013	1.212
***ant3***	6.075	4.404	***dfrA***	11.873	3.356	***qnrS***	0.000	0.000
***aph3***	4.902	1.460	***ermB***	0.398	0.067	***strA***	20.257	23.150
***blaACT***	4.955	2.093	***ermF***	0.029	0.025	***strB***	24.312	27.916
***blaCTX***	1.712	0.443	***floR***	0.160	0.038	***sul1***	3.752	0.310
***blaDHA***	27.260	3.069	***intl1***	8.956	40.562	***sul2***	15.770	26.838
***blaKPC***	0.014	0.000	***mcr1***	0.000	0.000	***sul3***	0.847	0.103
***blaNDM***	0.003	0.000	***mecA***	0.000	0.000	***tetA***	2.548	0.102
***blaSHV***	5.911	0.678	***oqxA***	10.975	10.701	***tetB***	3.619	6.323
***blaTEM***	28.909	38.144	***oqxB***	18.739	9.369	***tetM***	33.737	22.520
***blaVIM***	0.003	0.000				***vanA***	0.000	0.000

**Table 5:** Detection of 35 ARGs on ten locations in Norway using HT-qPCR on the Biomark Fluidigm 96.96 Dynamic DNA array. All ARG values above the threshold were normalized with respect to 16S. Locations A, B, D, and H were sampled both in 2019 (spring and autumn) and 2020 (spring), whereas the rest were only sampled in 2020 (spring).

Location	No. of Samples			%
	Total	16S-Positive	ARG-Positive	ARG-Positive
**A**	106	105	59	55.7
**B**	125	125	88	70.4
**C**	26	26	21	80.8
**D**	152	151	102	67.1
**E**	22	22	14	63.6
**F**	26	26	14	53.8
**G**	26	26	13	50.0
**H**	126	122	56	44.4
**I**	19	19	10	52.6
**J**	16	16	6	37.5
**Total sum**	**644**	**638**	**383**	**59.5**

## Data Availability

The reads were deposited in ENA under study accession number PRJNA1108478.

## Supplementary Materials

The following supporting information can be downloaded at https://www.mdpi.com/article/10.3390/microorganisms12091854/s1, Supplementary File S1: Table S1. Description of sampling locations and areas in 2019, Table S2. Number of samples collected in the spring 2019, Table S3. Number of samples collected in the autumn 2019, Table S4. Description of sampling locations and areas, as well as number of samples collected in 2020, Table S5. Antimicrobial agents included in phenotypic susceptibility testing of *E. coli*, Table S6. Assays designed for direct testing of 35 ARG (including *intl1*), using HT-qPCR, Table S7. Number of *E. coli* positive samples identified in 2019, Table S8. Phenotypic antimicrobial susceptibility testing of *E. coli* isolates (n = 313) using the EUVSEC panel from Sensititre® TREK, Table S9. Comparison of the prevalence of AREC positive samples within each of the variables Location, Season and Area type, calculated as the percentage of all samples, Table S10. Comparison of the prevalence of AREC positive samples within each of the variables Location, Season and Area type, calculated as the percentage of *E. coli* positive samples, Table S11. Comparison of results from WGS of antimicrobial resistant *E. coli* with results from a HT-qPCR detection of 35 ARG on the same *E. coli* isolates, as well as on the samples they were isolated from, Table S12. Element analysis of soil samples collected in the autumn 2019, Table S13A–C. Pesticides detected in soil samples Supplementary File S2: Figure S1. Standard curves QPCR assays, Supplementary File S3: Table S14. AMR46_assays-tehnical data, Supplementary File S4: Table S15. Year 2019, sample details and ARG results, Supplementary File S5: Table S16. Year 2020, sample details and ARG results.
