# Supplementary material for: Antimicrobial Resistance in the Terrestrial Environment of Agricultural Landscapes in Norway
Source: Microorganisms. 2024 Sep 6;12(9):1854. doi: 10.3390/microorganisms12091854 (PMC11433849; doi:10.3390/microorganisms12091854)
Supplement: Supplementary file 1 [file microorganisms-12-01854-s001.zip › Supplementary file 1 Tables S1 to S13.pdf]

Supplementary file 1

Table S1. Description of sampling locations and areas in 2019.

Location	Area type	Crops & vegetation	Manure used	Pesticides
A	Organic	Cereals	Cattle, poultry	
	Conventional	Cereals		Pesticides
	Control	Forest		
B	Organic	Grass	Grazing cattle, org. fertilizer	
	Conventional	Cereals		Pesticides
	Control	Forest		
D	Organic	Grass	Cattle	
	Conventional	Carrots	Cattle	Pesticides
	Control	Forest		
H	Organic	Grass	Cattle	
	Conventional	Carrots	Cattle	Pesticides
	Control	Forest		

Table S2. Number of samples collected in the spring 2019.

Location	Area type	Soil	Red clover	Slugs, snails	Mice, shrews	Earthworms	Sum area	Sum location
**A**	Organic	1	10			10	**21**	
	Conventional	1	10			10	**21**	
	Control	1					**1**	**43**
**B**	Organic	1	10			10	**21**	
	Conventional	1	10			10	**21**	
	Control	1					**1**	**43**
**D**	Organic	1	10	10	10		**31**	
	Conventional	1	10	10	2	8	**31**	
	Control	1					**1**	**63**
**H**	Organic	1	5	4		10	**20**	
	Conventional	1	5	10	1	10	**27**	
	Control	1					**1**	**48**
**Sum area type**	Organic	4	35	14	10	30	**93**	
	Conventional	4	35	20	3	38	**100**	
	Control	4					**4**	
**Total**		**12**	**70**	**34**	**13**	**68**	**197**	**197**

Table S3. Number of samples collected in the autumn 2019.

Location	Area type	Soil	Red clover	Slugs, snails	Mice, shrews	Sum area	Sum Location
**A**	Organic	1	10	10		**21**	
	Conventional	1	10	10	4	**25**	
	Control	1				**1**	**47**
**B**	Organic	1	10	10	3	**24**	
	Conventional	1	10	10	10	**31**	
	Control	1				**1**	**56**
**D**	Organic	1	10	10	10	**31**	
	Conventional	1	10	10	10	**31**	
	Control	1				**1**	**63**
**H**	Organic	1	10	10		**21**	
	Conventional	1	10	10	9	**30**	
	Control	1				**1**	**52**
**Sum area type**	Organic	4	40	40	13	**97**	
	Conventional	4	40	40	34	**118**	
	Control	4				**4**	
**Total**		**12**	**80**	**80**	**46**	**218**	**218**

Table S4. Description of sampling locations and areas, as well as number of samples collected in 2020.

Location	Area type	Crops & vegetation	Manure used	Pesticides used	Soil	Red clover	Slugs, snails	Sum area	Sum location
**A**	Organic	Cereals	Cattle, poultry		1	5		**6**	
	Conventional	Cereals		Pesticides	1	5		**6**	
	Control	Forest			4			**4**	**16**
**B**	Organic	Grass	Grazing cattle, org. fertilizer		1	5	5	**11**	
	Conventional	Cereals		Pesticides	1	5	5	**11**	
	Control	Forest			4			**4**	**26**
**C**	Organic	Grass	Cattle		1	5	5	**11**	
	Conventional	Clover		Pesticides	1	5	5	**11**	
	Control	Forest			4			**4**	**26**
**D**	Organic	Potato	Cattle		1	5	5	**11**	
	Conventional	Potato	Cattle	Pesticides	1	5	5	**11**	
	Control	Forest			4			**4**	**26**
**E**	Organic	Fruit	Poultry		1	5	1	**7**	
	Conventional	Fruit		Pesticides	1	5	5	**11**	
	Control	Forest, grass			4			**4**	**22**
**F**	Organic	Grass	Sheep		1	5	5	**11**	
	Conventional	Grass	Cattle	Glyphosate	1	5	5	**11**	
	Control	Forest, bog			4			**4**	**26**
**G**	Organic	Grass	Cattle		1	5	5	**11**	
	Conventional	Grass	Cattle	Herbicides	1	5	5	**11**	
	Control	Forest			4			**4**	**26**
**H**	Organic	Grass	Cattle		1	5	5	**11**	
	Conventional	Carrots	Cattle,	Pesticides	1	5	5	**11**	
	Control	Forrest			4			**4**	**26**
**I**	Organic	Grass			1	5	1	**7**	
	Conventional	Grass		Glyphosate	1	5	2	**8**	
	Control	Forest			4			**4**	**19**
**J**	Organic	Grass	Cattle		1	5		**6**	
	Conventional	Grass			1	5		**6**	
	Control	Forest			4			**4**	**16**
**Sum area type**	Organic				10	50	32	**92**	
	Conventional				10	50	37	**97**	
	Control				40			**40**	
**Total**					**60**	**100**	**69**	**229**	**229**

Table S5. Antimicrobial agents included in phenotypic susceptibility testing of *E. coli*.

Antimicrobial group	Antimicrobial agent
Tetracyclines	Tetracycline
	Tigecycline
Amphenicols	Chloramphenicol
Penicillins with extended spectrum	Ampicillin
Cephalosporins	Ceftazidime
	Cefotaxime
Carbapenems	Meropenem
Trimethoprim and derivates	Trimethoprim
Sulfonamides	Sulfamethoxazole
Macrolides	Azithromycin
Other aminoglycosides	Gentamicin
Fluoroquinolones	Ciprofloxacin
Other quinolones	Nalidixic acid
Polymyxins	Colistin

Table S6. Assays designed for direct testing of 35 ARG (including *intl1*), using HT-qPCR.

Gene	Antimicrobial class	No of assays
aac(3)	Aminoglycosides	1
aac(6)	Aminoglycosides	1
ant3	Aminoglycosides	2
aph3	Aminoglycosides	1
blaACT	Beta-lactams	1
blaCTX-M	Beta-lactams	2
blaDHA	Beta-lactams	1
blaKPC	Beta-lactams	1
blaNDM	Beta-lactams	2
blaSHV	Beta-lactams	1
blaTEM	Beta-lactams	1
blaVIM	Beta-lactams	3
catA	Amphenicols	1
cmlA	Amphenicols	1
dfrA	Dihydrofolate reductase inhibitors	1
ermB	Macrolides, lincosamides and streptogramines	3
ermF	Macrolides, lincosamides and streptogramines	2
floR	Amphenicols	1
intl1	Class 1 integron-integrase	1
mcr1	Colistin	1
mecA	Beta-lactams	1
oqxA	Quinolones	1
oqxB	Quinolones	1
qnrA1	Quinolones	1
qnrB1	Quinolones	1
qnrS	Quinolones	1
strA	Aminoglycosides	1
strB	Aminoglycosides	1
sul1	Sulphonamides	1
sul2	Sulphonamides	1
sul3	Sulphonamides	1
tetA	Tetracyclines	1
tetB	Tetracyclines	1
tetM	Tetracyclines	1
vanA	Glycopeptides	3
16S rRNA		1
**TOTAL**		**46**

Table S7. Number of *E. coli* positive samples identified in 2019. Aut. = Autumn, Org. = Organic, Conv. = Conventional, Contr. = Control

**Location**	**Area type***	**Soil**		**Red clover**		**Slugs, snails**		**Mice, shrews**		**Earth-worms**	**Sum area type**		**Sum location**	
		Spring	Aut.	Spring	Aut.	Spring	Aut.	Spring	Aut.	Spring	Spring	Aut.	Spring	Aut.
A	Org.	1	1	1	5		2			1	**3**	**8**		
	Conv.				3		1		4			**8**		
	Contr.	1									**1**		**4**	**16**
B	Org.	1		1	1		1		3		**2**	**5**		
	Conv.		1		1		1		10			**13**		
	Contr.	1	1								**1**	**1**	**3**	**19**
D	Org.	1	1	1	7	3	2	9	10		**14**	**20**		
	Conv.		1		3	2	1	2	6		**4**	**11**		
	Contr.		1									**1**	**18**	**32**
H	Org.	1	1	1			5				**2**	**6**		
	Conv.	1	1		2	2			3		**3**	**6**		
	Contr.	1	1								**1**	**1**	**6**	**13**
**Sum Organic**		**4**	**3**	**4**	**13**	**3**	**10**	**9**	**13**	**1**	**21**	**39**		
**Sum Conv.**		**1**	**3**	**0**	**9**	**4**	**3**	**2**	**23**	**0**	**7**	**38**		
**Sum Control**		**3**	**3**	**0**	**0**	**0**	**0**	**0**	**0**	**0**	**3**	**3**		
**Total**		**8**	**9**	**4**	**22**	**7**	**13**	**11**	**36**	**1**	**31**	**80**		

Table S8. Phenotypic antimicrobial susceptibility testing of *E. coli* isolates (n=313) using the EUVSEC panel from Sensititre® TREK. The isolates were from red clover (n=72), slugs/snails (n=52), mice/shrews (n=138), earthworms (n=3) and soil (n=48). Maximum three isolates may be from the same sample (mean 2.8 isolate per sample).

Substance	Resistance		Distribution (%) of MIC values (mg/L)*															
	(%)	[95% CI]	0.015	0.03	0.06	0.12	0.25	0.5	1	2	4	8	16	32	64	128	256	≥512
Tetracycline	0.3	[0 - 1.8]								96.2	2.6	1.0			0.3			
Tigecycline	0	[0 - 1.2]					99.7	0.3										
Chloramphenicol	4.5	[2.5 - 7.4]										87.9	7.7			0.6	3.8	
Ampicillin	4.8	[2.7– 7.8]							0.6	19.5	69.0	6.1		0.3		4.5		
Cefotaxime	0	[0 - 1.2]					100											
Ceftazidime	0	[0 - 1.2]						100										
Meropenem	0	[0 - 1.2]		100														
Trimethoprim	0.3	[0 - 1.8]										99.0	0.6					0.3
Sulfamethoxazole	0	[0 - 1.2]					39.0	53.7	7.0	0.3								
Azithromycin	**	**								4.5	48.9	43.8	2.9					
Gentamicin	0	[0 - 1.2]						87.9	10.2	1.9								
Ciprofloxacin	0	[0 - 1.2]	84.3	13.7	1.9													
Nalidixic acid	0	[0 - 1.2]									99.7	0.3						
Colistin	0	[0 - 1.2]							83.7	16.3								

* Bold vertical lines denote epidemiological cut-off values for resistance. CI = confidence interval. White fields denote range of dilutions tested for each antimicrobial agent. MIC values higher than the highest concentration tested are given as the lowest MIC value above the range. MIC values equal to or lower than the lowest concentration tested are given as the lowest concentration tested

** Cut-off value for azithromycin is not defined.

Table S9. Comparison of the prevalence of AREC positive samples within each of the variables Location, Season and Area type, calculated as the percentage of all samples.

Variable	Attribute / Percentage of samples with ABR			
Location	A / 4.4%	B / 1.0 %	D / 2.4 %	H / 0.0 %
Season, all sample types	Spring / 0.5 %	Autumn / 3.2 %		
Season, earthworms not included	Spring / 0.6 %	Autumn / 4.4 %		
Area type	Organic / 3.7 %	Conventional / 0.5 %	Control / 0.0 %	

* Chi square test, **Control area not included

Table S10. Comparison of the prevalence of AREC positive samples within each of the variables Location, Season and Area type, calculated as the percentage of *E. coli* positive samples.

Variable	Attribute / Percentage of *E. coli* positive samples with ABR			
Location	A / 20.0 %	B / 4.5 %	D / 6.0 %	H / 0.0 %
Season, all sample types	Spring / 3.2%	Autumn / 8.8 %		
Season, earthworms not included	Spring / 3.3 %	Autumn / 8.8 %		
Area type	Organic / 11.7 %	Conventional / 2.2 %	Control / 0.0 %	

* Chi square test, **Control area not included

Table S11. Comparison of results from WGS of antimicrobial resistant *E. coli* with results from a HT-qPCR detection of 35 ARG on the same *E. coli* isolates, as well as on the samples they were isolated from. ARG identified by WGS of the isolates are marked green. The HT-qPCR is a Biomark Fluidigm 96.96 Dynamic DNA array. All ARG values were normalized with respect to 16S. Values for 16S are given as ng/μL.

ID	Material	Genes and ARG values											
		16S	*intl1*	*aph3*	*blaTEM*	*catA*	*strA*	*strB*	*floR*	*sul2*	*tetA*	*tetB*	*tetM*
**1860-20**	**Sample**	0.922	1.965	0	3.681	0	2.236	2.141	0	2.465	1.547	0	0.023
**1860-20-2**	***E. coli* isolate**	0.455	4.904	0	6.269	0	5.258	8.977	0	5.644	3.145	0	0
**3544-20**	**Sample**	0.683	0	0	5.639	8.351	0	0	0	0	0	0	0
**3544-20-1**	***E. coli* isolate**	0.615	0	0	5.852	8.162	0	0	0	0	0	0	0
**3544-20-2**	***E. coli* isolate**	0.862	0	0	4.634	6.639	0	0	0	0	0	0	0
**3544-3**	**Sample**	0.744	0	0	2.428	2.720	0	0	0	0	0	0	0
**3544-3-1**	***E. coli* isolate**	0.854	0	0	4.529	6.690	0	0	0	0	0	0	0
**3544-3-2**	***E. coli* isolate**	1.068	0	0	3.177	4.534	0	0	0	0	0	0	0
**3544-5**	**Sample**	0.733	0	0.050	3.779	4.458	2.161	2.067	0	3.119	0	2.263	0
**3544-5-1**	***E. coli* isolate**	0.562	0	0	6.762	9.916	0	0	0	0	0	0	0
**3544-5-2**	***E. coli* isolate**	0.695	0	0	5.415	8.079	0	0	0	0	0	0	0
**3544-9**	**Sample**	0.655	0	0	3.755	3.965	0	0	0	0.018	0	0	0.151
**3544-9-1**	***E. coli* isolate**	0.553	0	0	6.084	8.922	0	0	0	0	0	0	0
**3544-9-2**	***E. coli* isolate**	0.756	0	0	4.918	7.170	0	0	0	0	0	0	0
**3820-1**	**Sample**	0											
**3820-1-1**	***E. coli* isolate**	1,036	0	0	0,0155	0	2,613	2,983	3,494	2,491	0	0	0
**3820-1-2**	***E. coli* isolate**	1,090	0	0	0,014	0	2,546	3,081	3,287	2,507	0	0	0
**3942-4**	**Sample**	0.507	0	0	0.762	0	0	0	0	0	0	0	0
**3942-4-3**	***E. coli* isolate**	1.027	0	0	3.643	0	0	0	0	0	0	0	0

Table S12. Element analysis of soil samples collected in the autumn 2019. Results are given as mean ± standard deviation mg/kg of four samples pr area type.

Element	Area type		
	Conventional	Organic	Control
Al	25062 ± 6097	20461 ± 5992	16936 ± 11964
As	4.65 ± 1.658	3.325 ± 1.269	2.325 ± 0.675
B	7.35 ± 5.48	6 ± 4.93	6.7 ± 4.21
Ba	172.1 ± 92.3	141.3 ± 72.1	138.3 ± 136.5
Be	0.95 ± 0.311	0.825 ± 0.15	0.65 ± 0.265
Ca	6888 ± 1727	8964 ± 7552	8617 ± 4386
Cd	1.725 ± 0.206	1.575 ± 0.427	1.375 ± 0.512
Co	8.78 ± 3.99	6.6 ± 1.523	5.63 ± 3.59
Cr	38.7 ± 19.42	28.43 ± 11.92	27.9 ± 25
Cu	16.88 ± 3.57	15.3 ± 3.32	14.9 ± 4.34
Fe	25615 ± 3421	22574 ± 7051	19327 ± 10365
Ga	10.2 ± 1.003	8.75 ± 3.1	7.9 ± 4.25
K	6559 ± 2403	4810 ± 1457	3776 ± 2209
Li	20.63 ± 9.85	14.37 ± 6.62	10.9 ± 9.96
Mg	6026 ± 2304	4564 ± 1068	4172 ± 2965
Mn	476.3 ± 162.4	500.5 ± 156.3	367 ± 311
Mo	0.4 ± 0	0.4 ± 0	0.7 ± 0.6
Na	405 ± 149.5	336.8 ± 83.9	415 ± 108.6
Ni	20.18 ± 15.34	12.77 ± 8.83	13.08 ± 13.75
P	1113 ± 303	1391 ± 215	1075.3 ± 64.4
Pb	13.28 ± 2.44	12.15 ± 3.55	17.5 ± 3.63
S	458 ± 289	326.2 ± 76	1296 ± 1469
Sc	6.7 ± 1.013	5.6 ± 1.08	5.55 ± 4.43
Se	2.2 ± 0	2.2 ± 0	2.2 ± 0
Sr	74.1 ± 5.82	82.1 ± 44.4	79.3 ± 31.8
Ti	216.8 ± 40.7	248 ± 72.7	237.8 ± 35.6
V	53.5 ± 18.15	40.52 ± 9.04	40.2 ± 28.3
Y	19.63 ± 2.98	18.05 ± 2.84	14.3 ± 9.3
Zn	65.95 ± 10.18	77 ± 20.9	48.3 ± 28

Table S13A. Pesticides detected in soil samples (0-5 cm depth) in areas under conventional cereal farming locations A and B in 2019. All concentrations are given as µg pesticide kg^{-1} dry soil. *F = fungicide, H = herbicide, I = insecticide, M = metabolite.*

**Pesticide**	***Type***	**Location**			
		A Spring	A Autumn	B Spring	B Autumn
**Propiconazole**	*F*			36.81	11.37
**Proquinazid**	*F*			6.86	
**Fludioxonil**	*F*	0.22	0.20	6.3	0.20
**Difenoconazole**	*F*			5.31	
**Prochloraz**	*F*	1.7	1.64	3.05	12.1
**Bixafen**	*F*			2.57	0.82
**Imazalil**	*F*			1.21	0.90
**Triticonazole**	*F*			1.16	4.37
**Fenpropidin**	*F*			0.77	0.75
**Azoxystrobin**	*F*	0.36	0.50	0.42	0.25
**Pyraclostrobin**	*F*	0.42	0.30	0.31	
**Mandipropamid**	*F*	5.82	3.33		
**Cyazofamid**	*F*	0.38	0.28		
**Trifloxystrobin**	*F*		1.05		
**Fluroxypyr**	*H*			20.85	
**Prosulfocarb**	*H*			11.39	0.18
**Diflufenican**	*H*			8.05	13.1
**Tribenuron-methyl**	*H*	0.24			0.21
**Metribuzin**	*H*	0.42	0.28		
**Proquinazidmet. IN-MM671**	*M*			2.38	
**Tribenuronmet. IN-L5296**	*M*		0.35	1.41	0.42
**Prothioconazole-desthio**	*M*	0.62	7.1	1.35	5.02
**Trifloxystrobinmet. CGA321113**	*M*	0.87	16.46	1.33	1.16
**Fluroxypyr-meptyl**	*M*	0.63	0.44	1.26	0.51
**Thiaclopridmet. M02**	*M*			0.41	1.5
**Tribenuronmet. IN-A4098**	*M*	0.37		0.37	1.2
**Metribuzinmet. DA**	*M*	0.41	0.38		
**Sum pesticides**		**12.7**	**32.5**	**113.6**	**54.1**

Table S13B. Pesticides detected in soil samples (0-5 cm depth) in areas under conventional carrot farming in location D and H in 2019. All concentrations are given as μg pesticide kg^{-1} dry soil. *F = fungicide, H = herbicide, I = insecticide, M = metabolite*.

**Pesticide**	***Type***	**Location**			
		D Spring	D Autumn	H Spring	H Autumn
**Boscalid**	*F*	18.78	259.56	21.08	156.68
**Pyraclostrobin**	*F*	0.96	23.22	0.58	13.58
**Mandipropamid**	*F*	1.37	0.80		
**Fludioxonil**	*F*	0.26	0.25		0.31
**Pencycuron**	*F*	0.19	0.17		
**Clomazone**	*H*	21.84	0.83	81.84	0.66
**Metribuzin**	*H*	14.68	0.36	25.59	0.56
**Linuron**	*H*	0.41	0.40	3.29	1.35
**Prosulfocarb**	*H*			0.23	0.14
**Lambda-cyhalothrin**	*I*			5.52	1.88
**Diazinon**	*I*			0.83	0.61
**Metribuzinmet. DA**	*M*	1.66	0.43	3.73	0.52
**Pyraclostrobinmet.**	*M*	1.28	5.18		3.15
**Pyridatmet. pyridafol**	*M*	0.77	0.49		
**Sum pesticides**		**62**	**292**	**147**	**179**

Table S13C. Pesticides detected in soil samples (0-5 cm depth) in areas under conventional farming in the counties included in the sampling in 2020. Results are only listed for counties with detectable residues and include locations with cereal (B), clover (C), potato (D), fruit production (E) and carrot (H). All concentrations are given as µg pesticide kg^{-1} dry soil. *F = fungicide, H = herbicide, I = insecticide, M = metabolite.*

**Pesticide**	***Type***	**Location**				
		B	C	D	E	H
**Boscalid**	*F*			4.94	102.61	45.97
**Fenhexamid**	*F*			73.46		
**Fenpropimorf**	*F*	0.74				
**Fluopyram**	*F*			66.57		
**Mandipropamid**	*F*		0.92		0.90	
**Penconazole**	*F*			90.55		
**Pencycuron**	*F*				44.79	
**Prochloraz**	*F*	8.11				
**Pyraclostrobin**	*F*				5.08	1.47
**Pyrimetanil**	*F*			33.56		
**Trifloxystrobin**	*F*	7.24				
**Triticonazole**	*F*	3.50				
**Clomazone**	*H*				0.56	0.54
**Diflufenican**	*H*	76.08				
**Isoxaben**	*H*			1.13		
**Linuron**	*H*					2.00
**Metribuzin**	*H*					49.21
**Indoxacarb**	*I*			55.26		
**Lambda-cyhalothrin**	*I*					20.82
**Spirodiclofen**	*I*	3.62				
**Spirotetramat**	*I*			8.48		
**Thiacloprid**	*I*			1.20		
**Boscalidmet. M510F49**	*M*				1.07	0.59
**Cyprodinil-met. CGA249287**	*M*		0.96			
**Fluopyram-7-OH**	*M*			2.73		
**Metribuzin-DA**	*M*					2.83
**Metsulfuron met. INA4098**	*M*	1.32				
**Prochloraz met. BTS44595**	*M*	5.32				
**Prothioconazole-desthio**	*M*	51.90				
**Pyracl.met.BF500-3**	*M*	1.23			0.61	
**Pyracl.met.BF500-6**	*M*				8.18	
**Pyridatmet. Pyridafol**	*M*				1.03	
**Spirotetramat-enol**	*M*			3.77		
**Spirotetramat-ketohydroxy**	*M*			33.19		
**Thiacloprid-amid**	*M*			5.60		
**Tribenuron met. INL5296**	*M*	1.13				
**Trifloxystrobin acid**	*M*	10.68				
**Sum pesticides**		**170.9**	**1.88**	**380.4**	**164.8**	**123.4**
