# Supplementary material for: Antimicrobial Resistance in the Terrestrial Environment of Agricultural Landscapes in Norway
Source: Microorganisms. 2024 Sep 6;12(9):1854. doi: 10.3390/microorganisms12091854 (PMC11433849; doi:10.3390/microorganisms12091854)

Supplementary Material B, Figure S1: Standard curves for 32 Assays

**16S_1**	
Slope	-3,340766223
R2	0,999627861
Efficiency	1,992198131
% E	99,21981308
CT max	24,4418051
Conc min	3,20E-05
CT min	5,235241475
Conc max	0,32

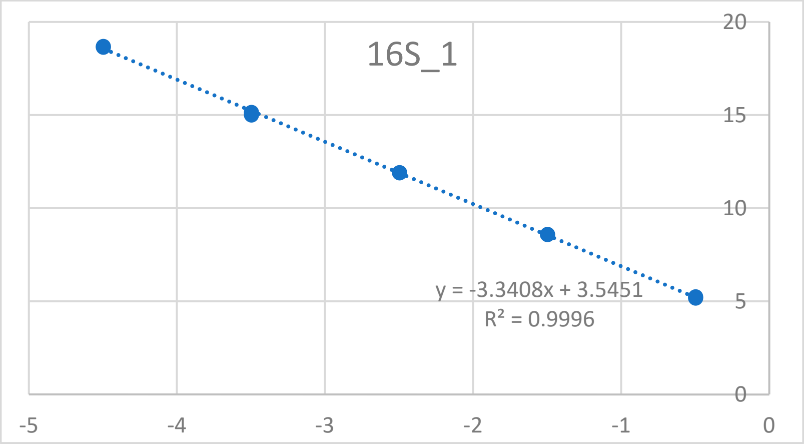

**AAC6_1**	
Slope	-3,39551864
R2	0,99942544
Efficiency	1,97017961
% E	97,0179608
CT max	24,3413903
Conc min	3,40E-06
CT min	7,5479923
Conc max	0,34

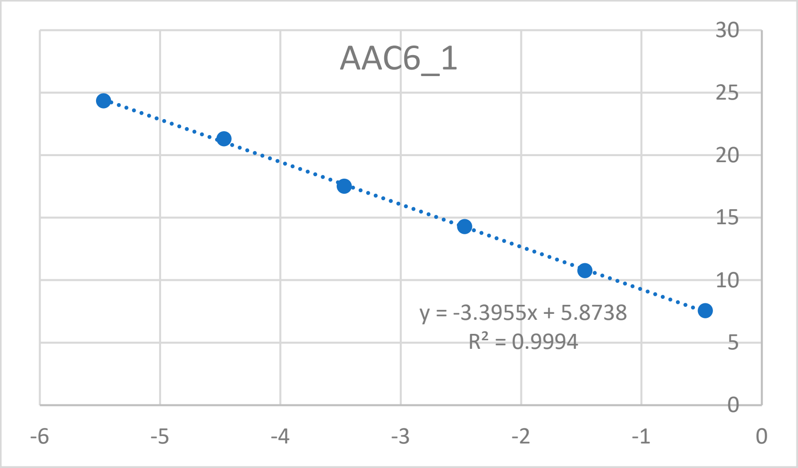

**AAC3_2**	
Slope	-3,62375373
R2	0,99912791
Efficiency	1,88780418
% E	88,7804177
CT max	18,3865753
Conc min	7,30E-03
CT min	11,2033671
Conc max	0,73

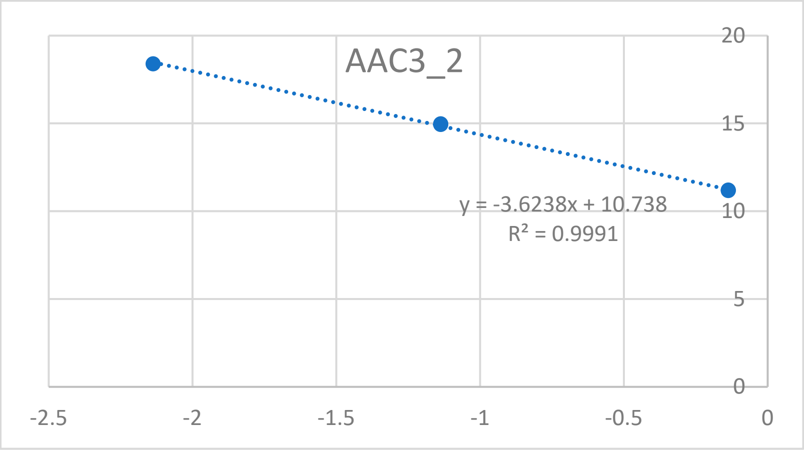

Supplementary Material B, Figure S1: Standard curves for 32 Assays

**Ant3_1**	
Slope	-3,33076844
R2	0,99864477
Efficiency	1,99632395
% E	99,6323954
CT max	23,4524328
Conc min	3,40E-06
CT min	6,91131798
Conc max	0,34

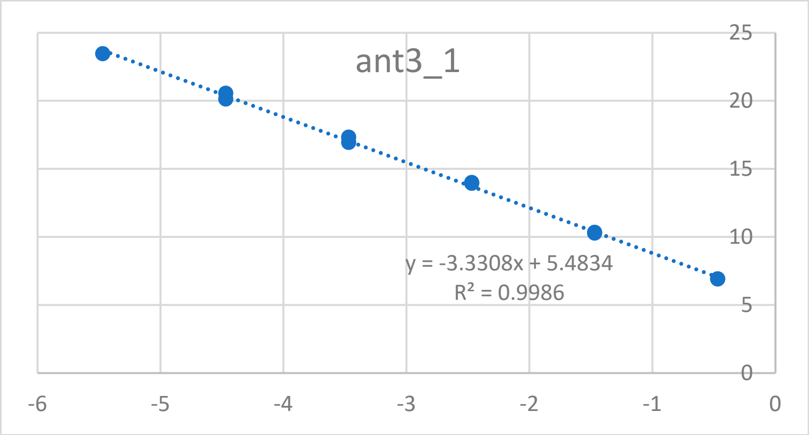

**Ant3_2**	
Slope	-3,37039032
R2	0,99946835
Efficiency	1,98016573
% E	98,0165734
CT max	17,5858125
Conc min	7,30E-04
CT min	10,7483902
Conc max	0,073

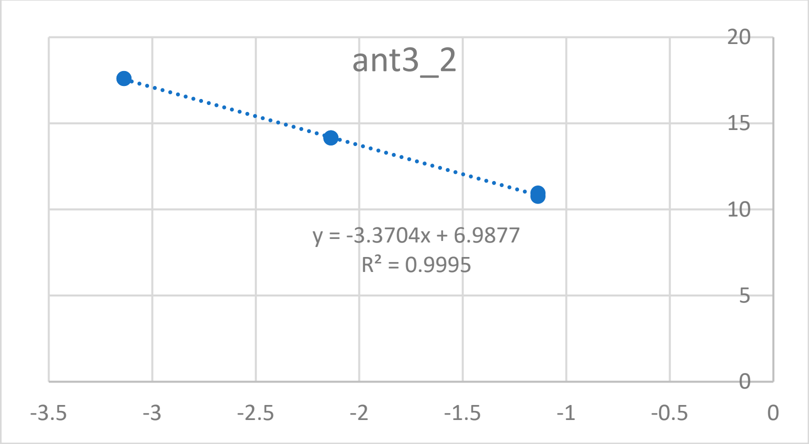

**Aph3_2**	
Slope	-3,41601311
R2	0,99693282
Efficiency	1,96218036
% E	96,2180355
CT max	21,0407258
Conc min	7,30E-05
CT min	7,54193389
Conc max	0,73

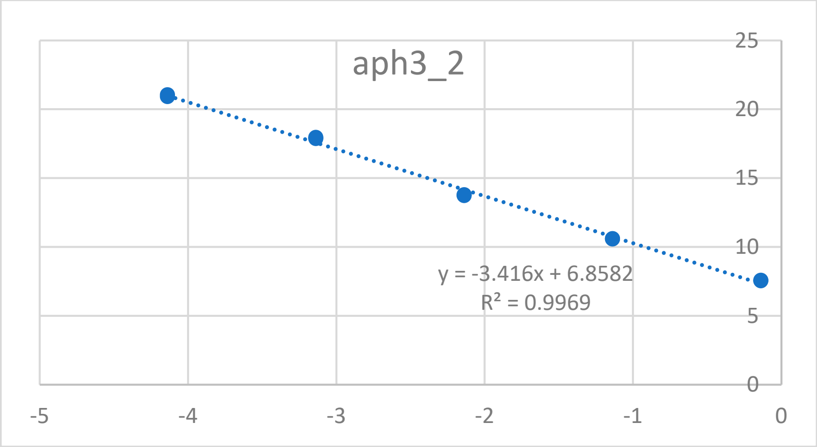

Supplementary Material B, Figure S1: Standard curves for 32 Assays

**blaACT_3**	
Slope	-3,74131897
R2	0,99919936
Efficiency	1,85048435
% E	85,048435
CT max	19,3907503
Conc min	3,10E-04
CT min	11,9605133
Conc max	0,031

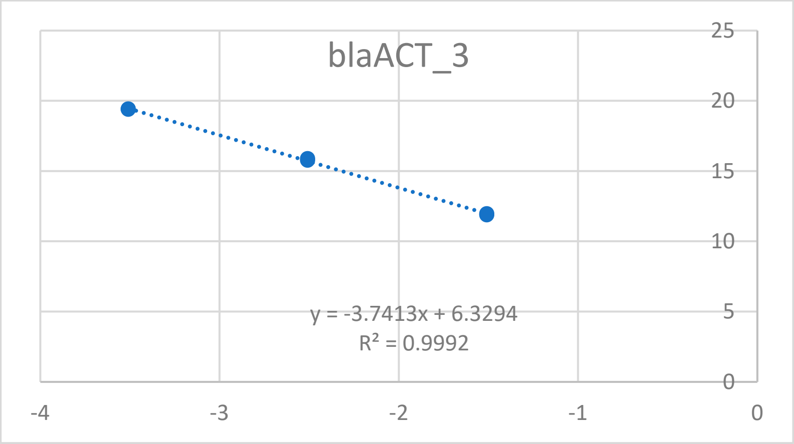

**blaCTX_2**	
Slope	-3,37596497
R2	0,9998964
Efficiency	1,97793313
% E	97,7933127
CT max	18,5591985
Conc min	3,20E-04
CT min	8,31894203
Conc max	0,32

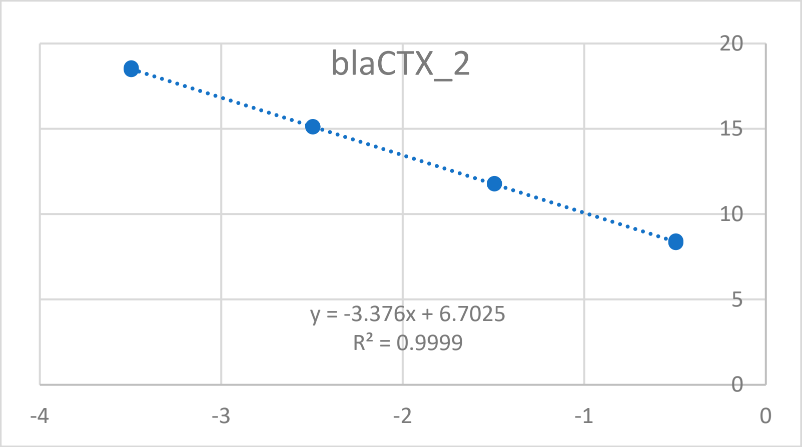

**blaCTX_3**	
Slope	-3,31505948
R2	0,99668139
Efficiency	2,00287439
% E	100,287439
CT max	21,9211842
Conc min	3,70E-05
CT min	8,88457116
Conc max	0,37

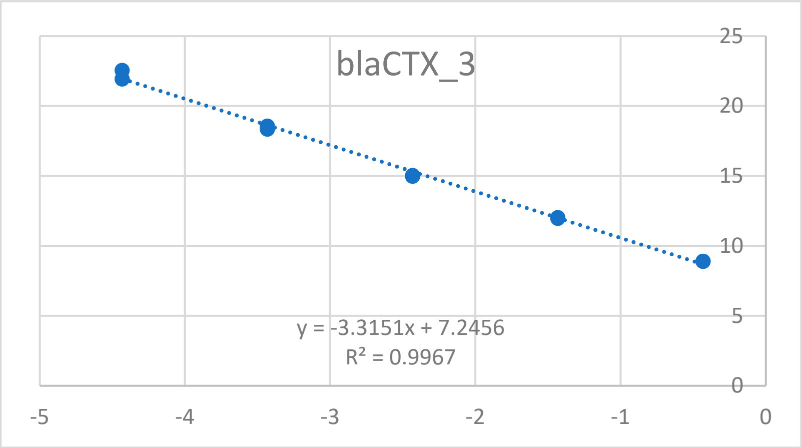

Supplementary Material B, Figure S1: Standard curves for 32 Assays

**blaDHA_1**	
Slope	-3,48117799
R2	0,99927765
Efficiency	1,9375775
% E	93,7577504
CT max	19,4611946
Conc min	1,00E-05
CT min	5,7062245
Conc max	0,1

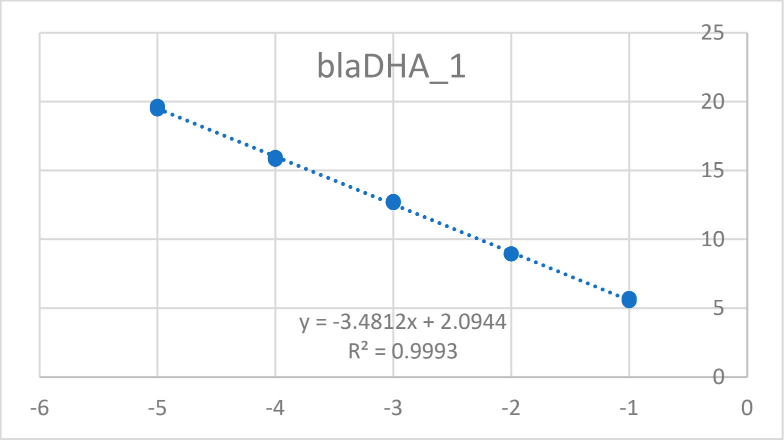

**blaKPC_1**	
Slope	-3,27212076
R2	0,9984194
Efficiency	2,02121351
% E	102,121351
CT max	21,0205431
Conc min	9,42E-06
CT min	11,2752656
Conc max	0,00942

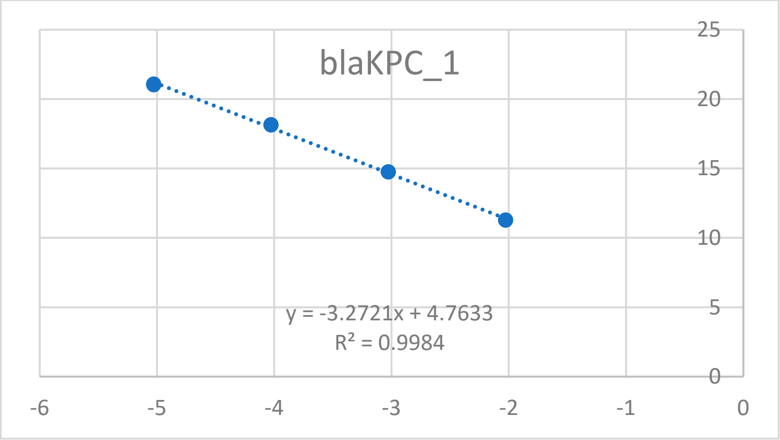

**blaSHV_1**	
Slope	-3,49504688
R2	0,98189376
Efficiency	1,93249863
% E	93,2498631
CT max	23,1811202
Conc min	4,50E-05
CT min	8,74675428
Conc max	0,45

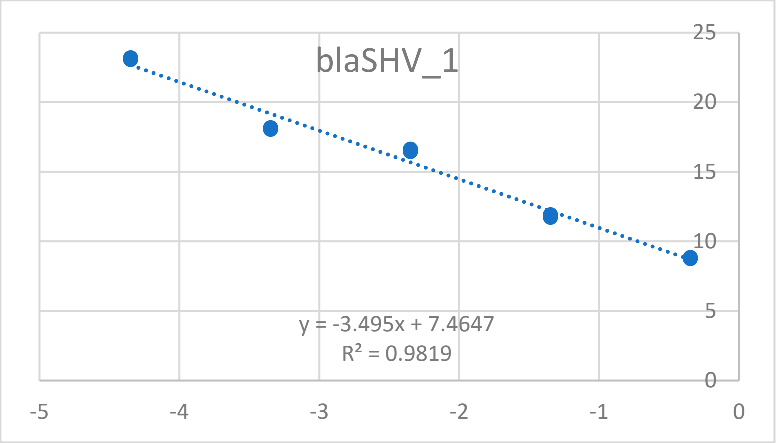

Supplementary Material B, Figure S1: Standard curves for 32 Assays

**blaTEM_1**	
Slope	-3,204009192
R2	0,99960517
Efficiency	2,051676893
% E	105,1676893
CT max	22,24779801
Conc min	3,70E-05
CT min	9,420641073
Conc max	0,37

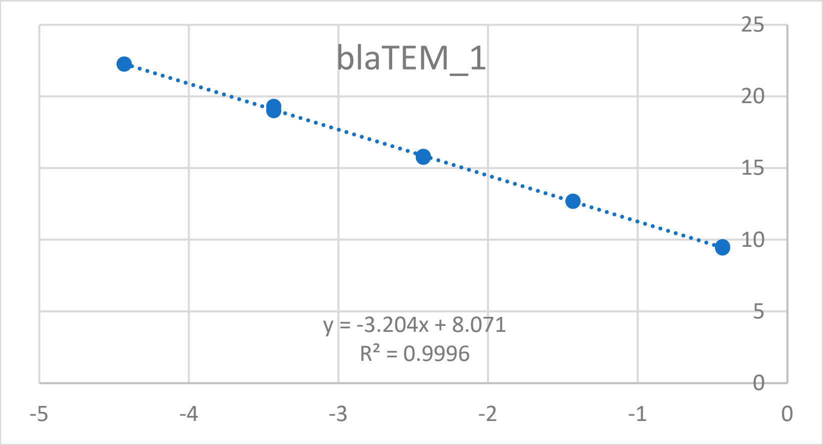

**catA_2**	
Slope	-3,35541654
R2	0,99976318
Efficiency	1,98621196
% E	98,6211957
CT max	18,8326967
Conc min	7,30E-04
CT min	8,70318237
Conc max	0,73

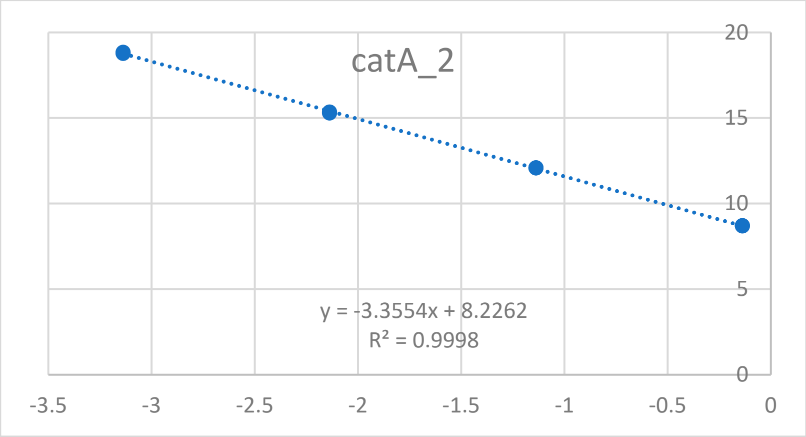

**cmlA_3**	
Slope	-3,31108231
R2	0,98251481
Efficiency	2,00454611
% E	100,454611
CT max	21,0086242
Conc min	4,50E-05
CT min	8,36947892
Conc max	0,45

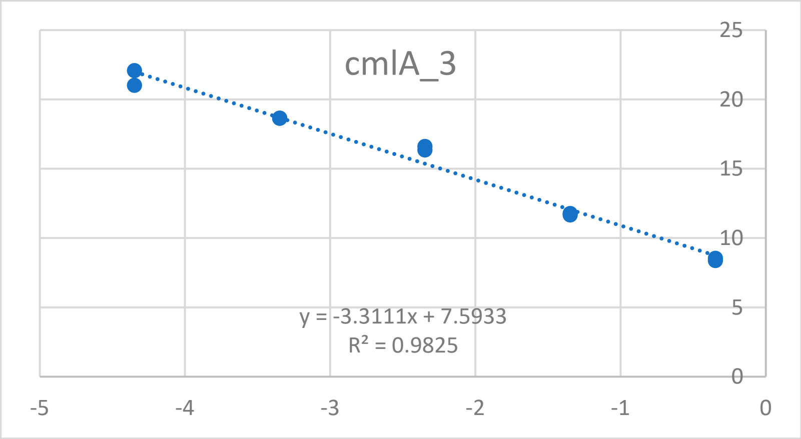

Supplementary Material B, Figure S1: Standard curves for 32 Assays

Slope	-3,16702603
R2	0,9949872
Efficiency	2,06896737
% E	106,896737
CT max	22,132344
Conc min	3,70E-05
CT min	8,92100605
Conc max	0,37

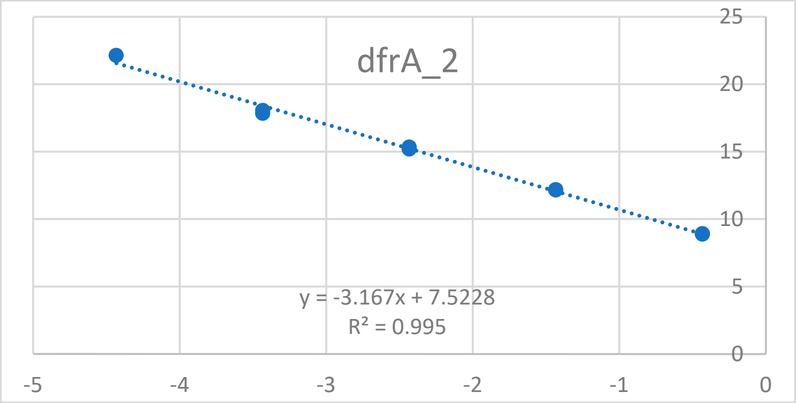

**floR_2**	
Slope	-3,35579208
R2	0,98624142
Efficiency	1,98605943
% E	98,6059432
CT max	21,8692992
Conc min	6,30E-05
CT min	9,03167685
Conc max	0,63

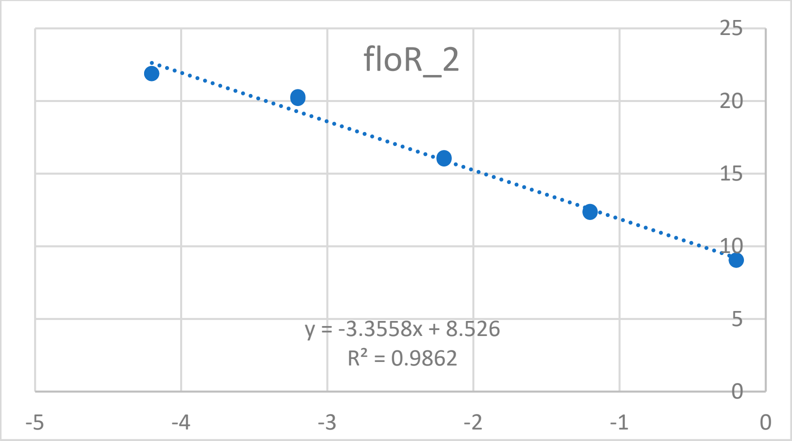

**Intl1_1**	
Slope	-3,3432032
R2	0,99980445
Efficiency	1,99119748
% E	99,1197483
CT max	17,9156444
Conc min	3,20E-04
CT min	7,80619079
Conc max	0,32

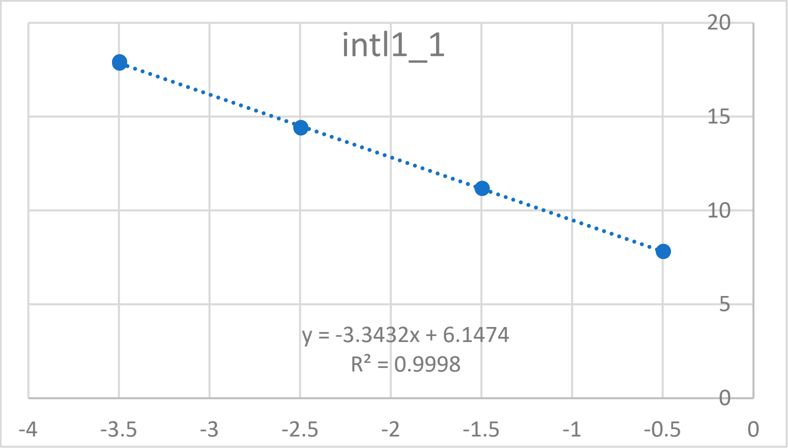

Supplementary Material B, Figure S1: Standard curves for 32 Assays

**mcr1_2**	
Slope	-3,38033909
R2	0,98917281
Efficiency	1,97618824
% E	97,6188238
CT max	20,1814824
Conc min	6,30E-05
CT min	6,99666587
Conc max	0,63

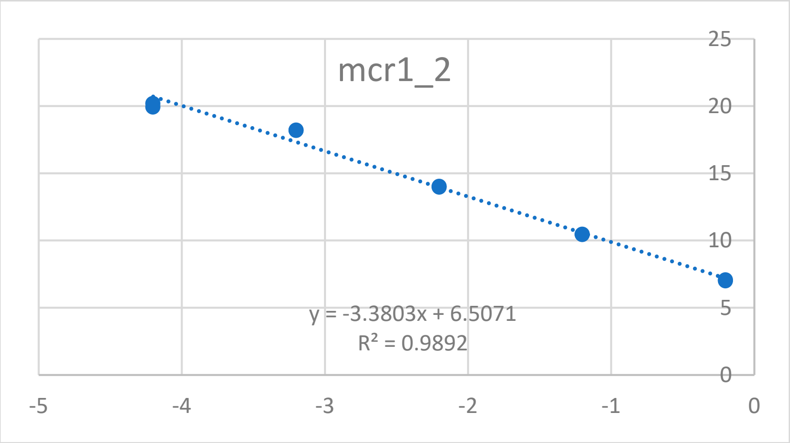

**mecA_1**	
Slope	-4,02933692
R2	0,99976888
Efficiency	1,77084191
% E	77,0841913
CT max	23,0215029
Conc min	2,20E-05
CT min	14,9132202
Conc max	0,0022

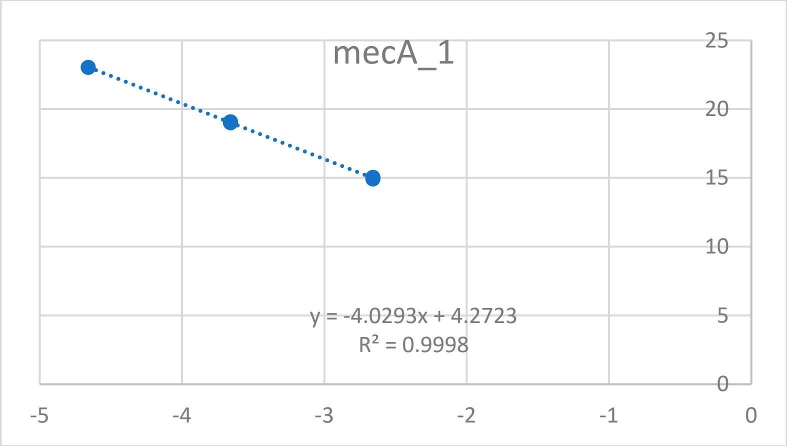

**oqxA_3**	
Slope	-3,33438015
R2	0,99858094
Efficiency	1,99482966
% E	99,4829657
CT max	20,9387143
Conc min	2,20E-04
CT min	10,8410121
Conc max	0,22

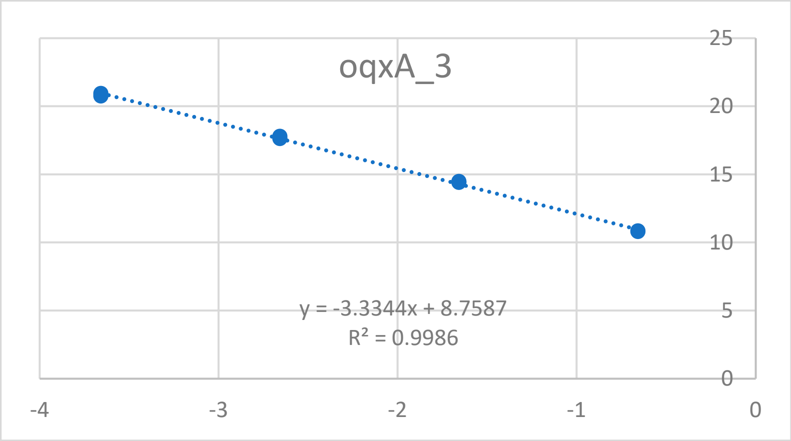

Supplementary Material B, Figure S1: Standard curves for 32 Assays

**oqxB_1**	
Slope	-3,28513022
R2	0,99811636
Efficiency	2,0155888
% E	101,55888
CT max	19,565833
Conc min	2,20E-04
CT min	9,55633028
Conc max	0,22

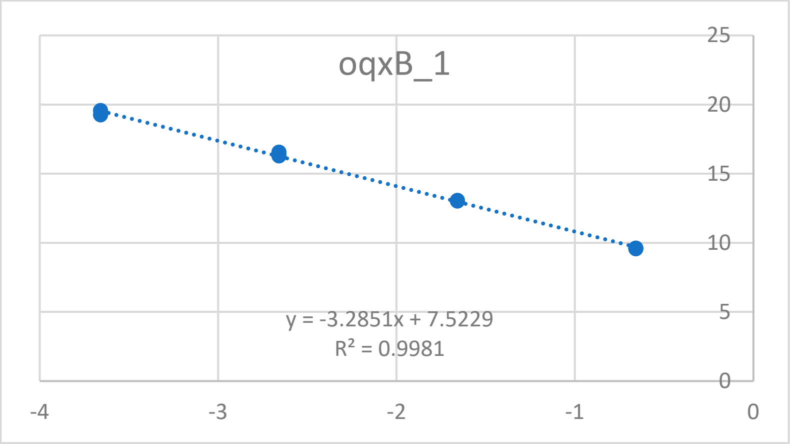

**qnrB1_2**	
Slope	-3,40212298
R2	0,98135533
Efficiency	1,96758776
% E	96,7587765
CT max	21,2505061
Conc min	3,70E-05
CT min	10,7360121
Conc max	0,037

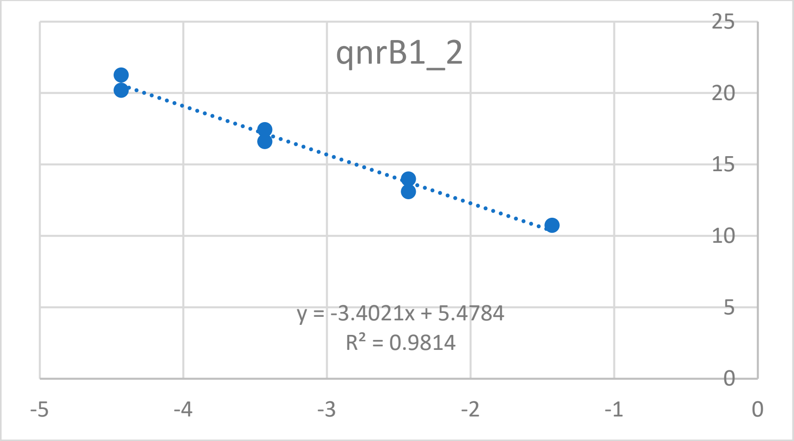

**qnrS_1**	
Slope	-3,46059528
R2	0,99986138
Efficiency	1,94521507
% E	94,5215069
CT max	18,9457156
Conc min	2,20E-04
CT min	8,51881396
Conc max	0,22

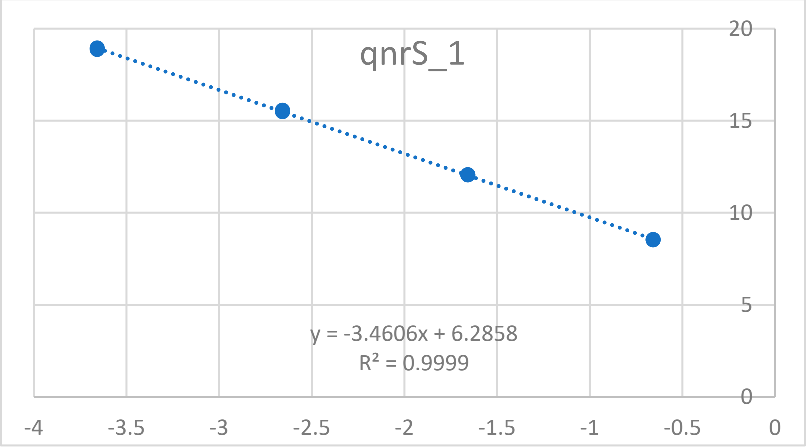

Supplementary Material B, Figure S1: Standard curves for 32 Assays

**strA_3**	
Slope	-3,2615968
R2	0,99777424
Efficiency	2,02580803
% E	102,580803
CT max	18,1624957
Conc min	3,40E-04
CT min	8,39869908
Conc max	0,34

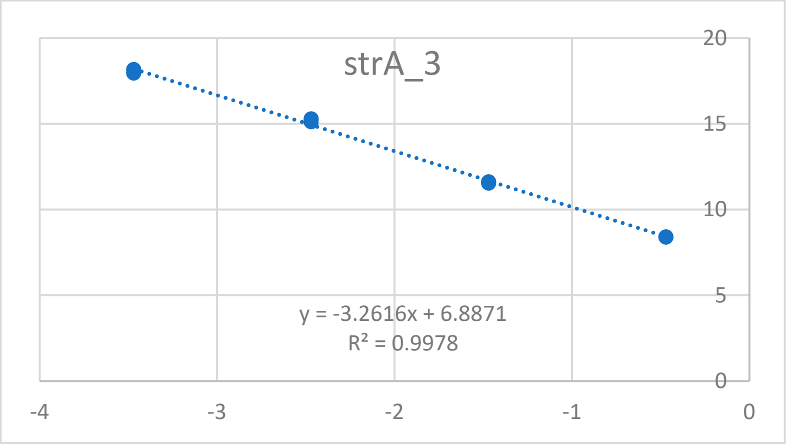

**strB_2**	
Slope	-3,37931856
R2	0,99957699
Efficiency	1,9765948
% E	97,6594798
CT max	17,7917675
Conc min	7,30E-04
CT min	7,63045039
Conc max	0,73

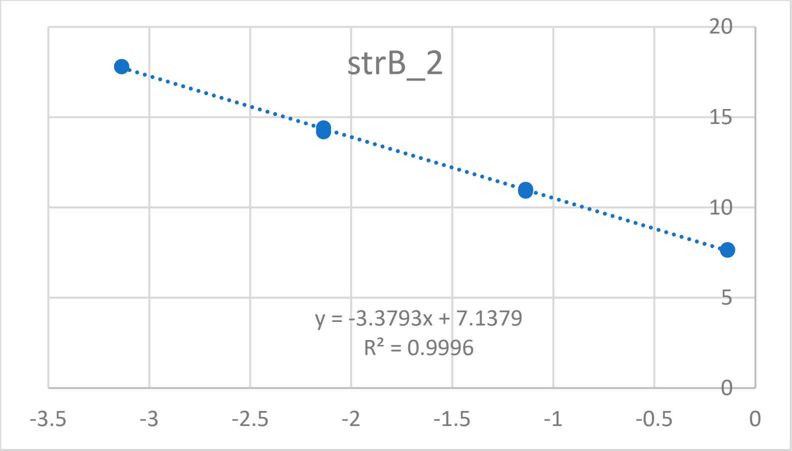

**sul1_3**	
Slope	-3,37462454
R2	0,99718123
Efficiency	1,97846906
% E	97,8469056
CT max	21,0713533
Conc min	3,40E-05
CT min	7,61195886
Conc max	0,34

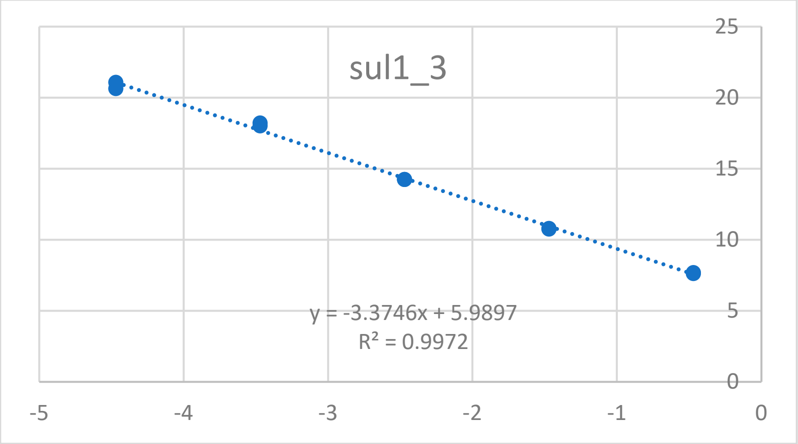

Supplementary Material B, Figure S1: Standard curves for 32 Assays

**sul2_2**	
Slope	-3,28930832
R2	0,99637684
Efficiency	2,01379512
% E	101,379512
CT max	21,4842189
Conc min	3,40E-05
CT min	7,83495311
Conc max	0,34

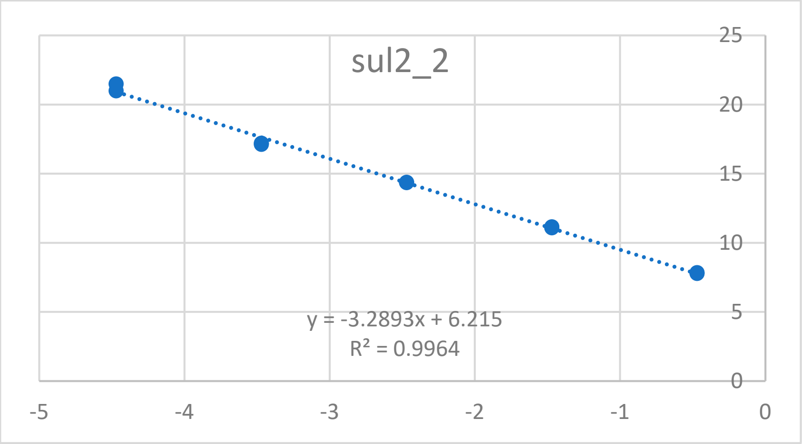

**sul3_3**	
Slope	-3,56626674
R2	0,9817975
Efficiency	1,90723968
% E	90,723968
CT max	21,9985776
Conc min	4,50E-05
CT min	7,49563956
Conc max	0,45

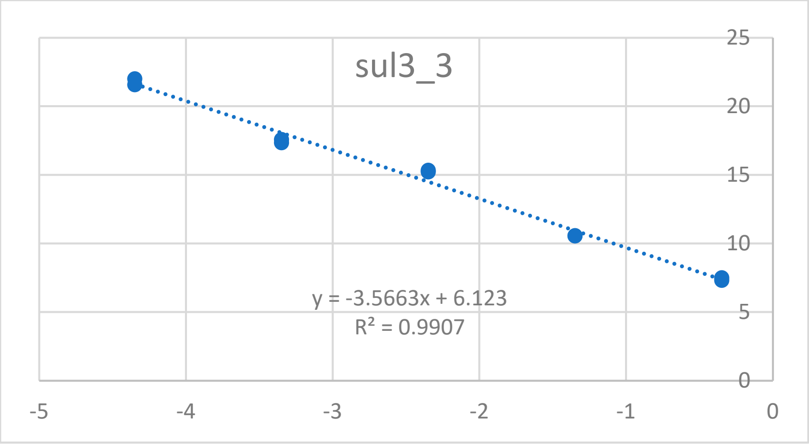

**tetA_3**	
Slope	-3,49000832
R2	0,99243531
Efficiency	1,93433758
% E	93,4337576
CT max	21,2690948
Conc min	6,30E-05
CT min	7,52967588
Conc max	0,63

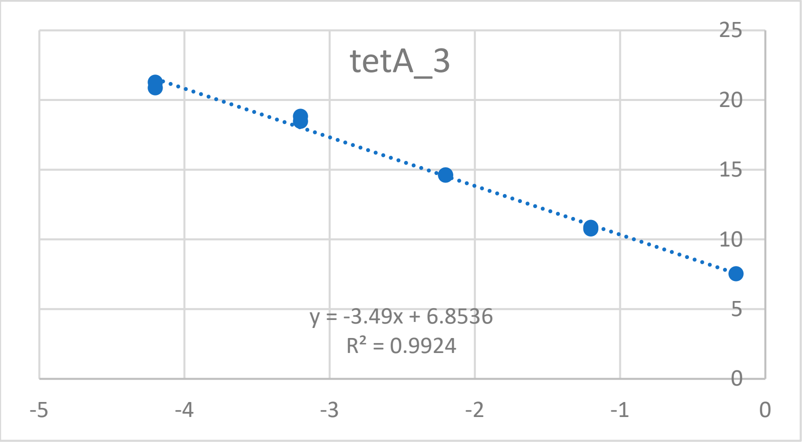

Supplementary Material B, Figure S1: Standard curves for 32 Assays

**tetB_2**	
Slope	-3,38369333
R2	0,99950038
Efficiency	1,97485428
% E	97,4854284
CT max	21,3894995
Conc min	7,30E-05
CT min	7,85817483
Conc max	0,73

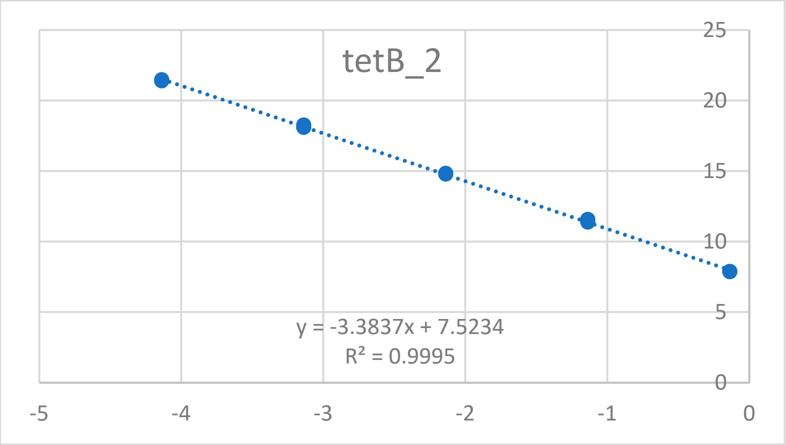

**tetM_2**	
Slope	-3,40711502
R2	0,99397321
Efficiency	1,96563758
% E	96,5637579
CT max	20,6357273
Conc min	6,30E-05
CT min	7,59214758
Conc max	0,63

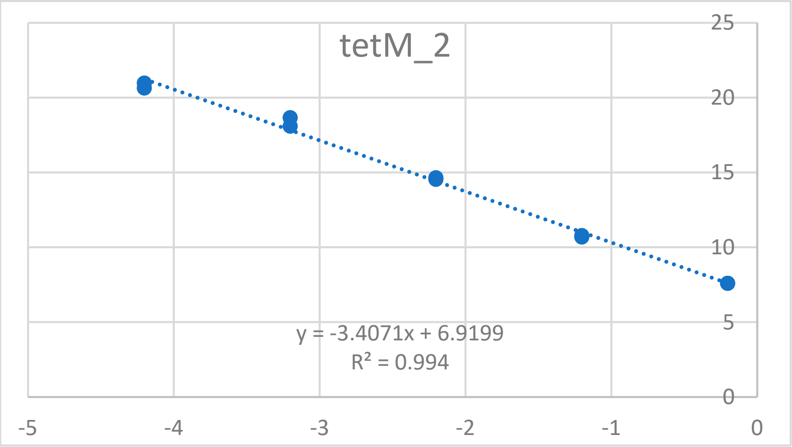

Supplement: Supplementary file 1 [file microorganisms-12-01854-s001.zip › Supplementary file 2 Figure S1 Standard curves QPCR assays.pdf]
